# Virulence hierarchies within the *Mycobacterium tuberculosis* complex

**DOI:** 10.1073/pnas.2507104122

**Published:** 2025-10-16

**Authors:** Sarah N. Danchuk, Shannon C. Duffy, Jaryd Sullivan, Syed Beenish Rufai, Fiona A. McIntosh, Andréanne Lupien, Luke B. Harrison, Hojjat Ghasemi Goojani, Lorne Taylor, Yuhong Wei, Philippe Joubert, Rasmus Mortensen, Jeffrey M. Chen, Nirajan Niroula, Robin Stevens, Carla Norleen, Vivek Kapur, Marcel A. Behr

**Affiliations:** ^a^Department of Microbiology and Immunology, McGill University, Montreal, QC H3A 0G4, Canada; ^b^Infectious Disease and Immunity in Global Health Program, Research Institute of the McGill University Health Centre, Montreal, QC H4A 0B1, Canada; ^c^McGill International Tuberculosis Centre, Montreal, QC H4A 3S5, Canada; ^d^Department of Epidemiology of Microbial Diseases, Yale School of Public Health, New Haven, CT 06510; ^e^Department of Molecular Biology and Centre for Computational and Integrative Biology, Massachusetts General Hospital, Boston, MA 02114; ^f^Department of Neuroscience, University of Lethbridge, Lethbridge, AB T1K 3M4, Canada; ^g^Department of Biochemistry and Medical Genetics, University of Manitoba, Winnipeg, MB R3T 2N2, Canada; ^h^Department of Medicine, McGill University, Montreal, QC H3A 0G4, Canada; ^i^Goodman Cancer Institute, McGill University, Montreal, QC H3A 0G4, Canada; ^j^Department of Pathology and Cytology, Institut Universitaire de Cardiologie et de Pneumologie de Québec-Laval University, Quebec City, QC G1V 4G5, Canada; ^k^Center for Vaccine Research, Department of Infectious Disease Immunology, Statens Serum Institut, Copenhagen 2300, Denmark; ^l^Mycobacterial Pathogenesis and Tuberculosis Research Laboratory, Vaccine and Infectious Disease Organization, University of Saskatchewan, Saskatoon, SK S7N 5E3, Canada; ^m^Department of Animal Science and the Huck Institutes of the Life Sciences, The Pennsylvania State University, University Park, PA 16802

**Keywords:** tuberculosis, pathogenesis, virulence, *Mycobacterium tuberculosis* complex

## Abstract

The *Mycobacterium tuberculosis* complex (MTBC) includes *M. tuberculosis* (*M. tb*), the primary cause of human tuberculosis, *M. bovis,* the classical zoonotic pathogen and cause of bovine tuberculosis, and *M. orygis,* a recently recognized multihost pathogen. Given that *M. tb, M. bovis,* and *M. orygis* pose significant threats to the health of humans and animals, we sought to understand fundamental differences in pathogenicity among these closely related organisms. Building upon historical observations, we conducted a comparative virulence assessment of these pathogens using both bovine and murine infection models. Holstein calves were infected via aerosol with *M. tb, M. bovis,* or *M. orygis*, and pathology was analyzed through macroscopic and microscopic assessments of lungs and lymph nodes, along with quantitative tissue bacterial burden measurements. In C57BL/6 mice, we compared virulence using three readouts, namely survival, lethal dose determination, and detailed pathological assessments. Despite genomic similarity, animal-adapted MTBCs consistently showed dramatically enhanced virulence compared to *M. tb* with distinct immunopathology and, in the murine model, mortality within 24 days. Using gene disruption studies guided by proteomic comparisons, we determined that these infection outcomes were dependent on shared (ESAT-6) and lineage-associated (MPT70) virulence factors, the route of infection, and prior infection or immunization. Our findings reveal unexpected virulence hierarchies within the MTBC, with fundamental and translational implications for tuberculosis research.

## Significance

This work challenges the conventional understanding of virulence within the *Mycobacterium tuberculosis* complex (MTBC). By directly comparing *M. tb, M. bovis*, and *M. orygis*, we demonstrate that animal-adapted MTBCs (*M. bovis* and *M. orygis*) show dramatically enhanced virulence compared to *M. tb*. Further, comparative proteomics and targeted gene-disruption demonstrate that this differential virulence is due to shared and lineage-specific factors. We observed a route-dependent phenomenon where *M. orygis* causes severe disease via aerosol exposure at low doses yet induces protective immunity following high-dose oral challenge. These findings advance our understanding of mycobacterial pathogenesis, suggest new opportunities for host-tailored, pathogen-specific vaccine development, and provide evidence for a new virulence hierarchy within the MTBC.

The *Mycobacterium tuberculosis* complex (MTBC) comprises a group of closely related pathogens that cause tuberculosis in mammalian hosts. Despite sharing >99.9% nucleotide identity, these organisms have distinct host specificities and can be broadly categorized into either the human-associated lineages (*M. tuberculosis sensu stricto* and *M. africanum*) or the animal-associated lineages (1). The latter include *M. bovis,* recognized globally as the primary cause of bovine tuberculosis, and *M. orygis*, recently recognized as an important multihost pathogen and cause of tuberculosis in both livestock and humans across South Asia (*SI Appendix*, Fig. S1) (1, 2).

Despite more than a century of research on MTBC members, direct comparisons of virulence in standardized experimental models remain surprisingly rare (3–8). The few comparative studies suggest that *M. bovis* exhibits enhanced virulence—also known as pathogenic potential—compared to *M. tuberculosis* across multiple species (3–8). However, the molecular mechanisms underlying these virulence differences are poorly understood. In the case of the emerging pathogen, *M. orygis*, our knowledge is even more limited, as it was only officially recognized in 2024 as a cause of zoonotic tuberculosis (zTB), and its epidemiological consequence has yet to be fully realized (9–12).

To address these knowledge gaps, we conducted systematic comparative analyses of *M. tb, M. bovis,* and *M. orygis* in two systems: cattle (a natural host) and mice (a well-characterized laboratory model). Our findings indicate that *M. bovis* and *M. orygis* show greater virulence compared to *M. tb*, in both hosts. Further, the outcome of infection is influenced by both conserved and lineage-specific virulence determinants, the route of exposure, and immunological history. These insights into mycobacterial pathogenesis suggest new opportunities for preventing tuberculosis through targeted immunization strategies and may have important implications for zoonotic control.

## Results

### Differential Virulence in the Calf Model.

**Fig. 1.**
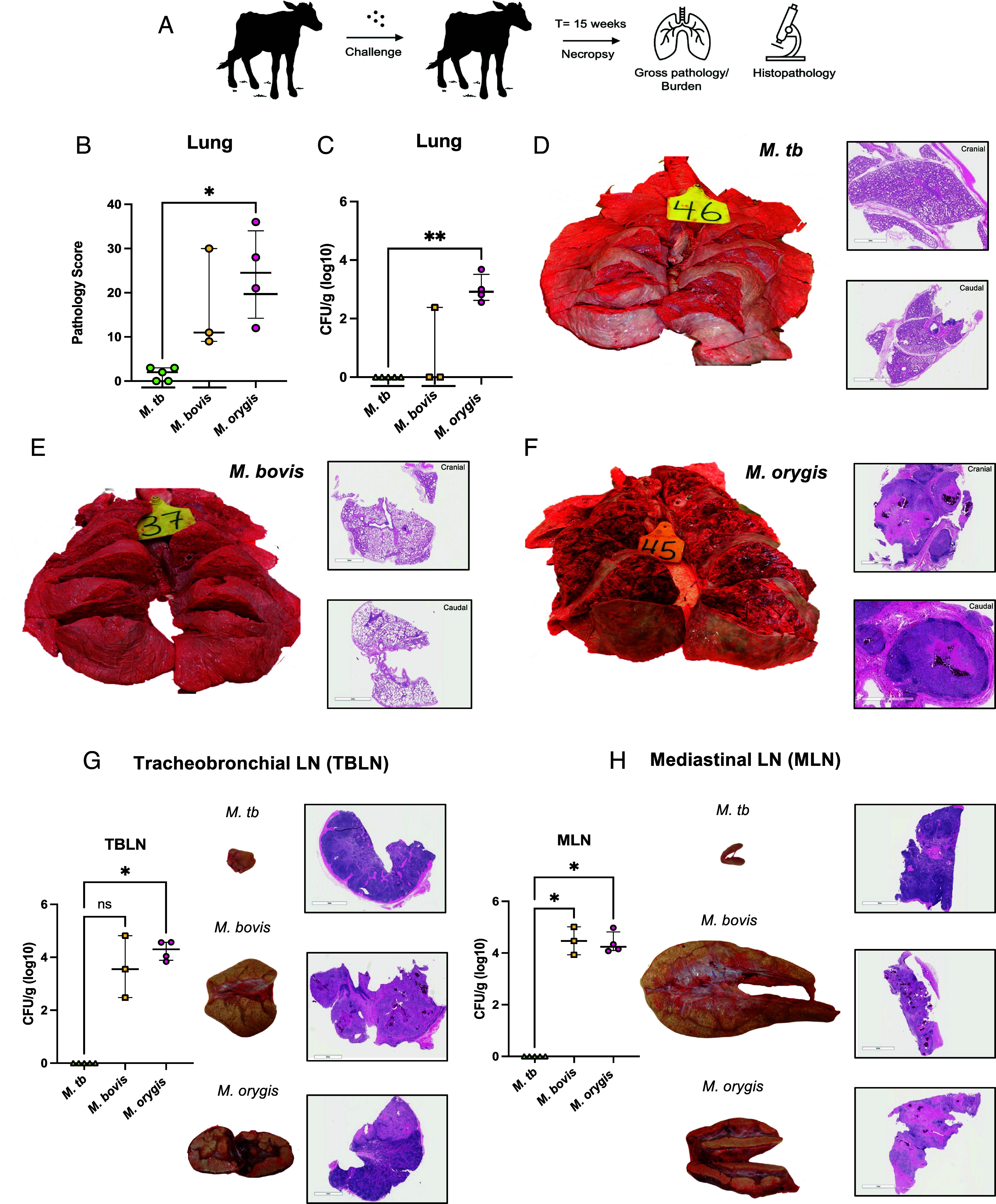
Bovine infection outcomes reveal differential virulence between MTBC members. (*A*) Experimental overview. *Bos taurus* calves were infected with *M. tb* H37Rv, *M. bovis* AF2122/97, or *M. orygis* 51145 (~10^4^ CFU/calf). At week 15, organs were harvested for bacterial burden and pathology. (*B*) Pathology scores of *M. tb, M. bovis,* and *M. orygis–*infected calves at endpoint. (*C*) Lung bacterial burden (CFU/g) calculated as sum of left + right cranial, caudal, and middle lobes. (*D*) *Left*: gross pathology of *M. tb–*infected lungs with the highest pathology score (3/40); *Right*: histopathology corresponding to *M. tb–*infected lungs (cranial and caudal lobes). (*E*) *Left*: gross pathology of *M. bovis–*infected lungs with the highest pathology score (30/40); *Right*: corresponding histopathology (cranial and caudal lobes). (*F*) *Left*: gross pathology of *M. orygis–*infected lungs with the highest pathology score (36/40); *Right*: corresponding histopathology (cranial and caudal lobes). (*G*) *Left*: bacterial burden of *M. tb, M. bovis,* and *M. orygis–*infected tracheo bronchial lymph nodes (TBLNs) calculated as CFU/g. *Middle*: gross pathology of *M. tb, M. bovis,* and *M. orygis–*infected TBLNs. All gross pathology representative of calves with the highest lung gross pathology score (Fig. 1 *D*–*F*). *Right*: corresponding histopathology of TBLNs. (*H*) *Left*: bacterial burden of *M. tb, M. bovis,* and *M. orygis–*infected mediastinal lymph node (MLN) (CFU/g). *Middle*: gross pathology of *M. tb, M. bovis,* and *M. orygis–*infected MLNs. All gross pathology representative of calves with highest lung gross pathology score (Fig. 1 *D–F*). *Right*: corresponding histopathology of MLNs. Comparisons by Kruskal–Wallis test, * indicates *P* < 0.05, ** indicates *P* < 0.01.

Fifteen calves (5 per group) were infected via the aerosol route with either *M. tb*, *M. bovis*, or *M. orygis* (10^4^ colony-forming units) (Fig. 1*A*). Three calves (two *M. bovis*, one *M. orygis*) failed to reach experimental endpoint (15 wk postinfection) due to acute bloat (n = 2) or acute pneumonia (n = 1). Of the remaining 12 calves, weight did not differ between experimental groups.

At necropsy, the five *M. tb*–infected calves showed the lowest gross pathology scores, limited evidence of granulomas microscopically, and undetectable bacterial burden (Fig. 1 *B*–*D*). In stark contrast, the remaining *M. bovis* and *M. orygis*–infected calves presented with visible and palpable lesions, shown on hematoxylin & eosin (H&E) staining to reveal a gradation of granulomas along with extensive necrotic foci (Fig. 1 *E* and *F* and *SI Appendix*, Fig. S2 *A*–*C*). Further, *M. orygis*–infected calves presented with greater pathology and bacterial burden compared to the *M. bovis* group; bacteria could be detected in the lungs of all 4 *M. orygis* calves (a 3-log_10_ difference compared to the *M. tb* group) but only a single *M. bovis*–infected calf (Fig. 1*C*).

Consistent with lung findings, *M. tb* showed no evidence of lymphatic disease. Comparatively, *M. bovis* and *M. orygis* both presented with significantly enlarged tracheobronchial lymph nodes (TBLN) and mediastinal lymph nodes (MLN), with varying degrees of hemorrhage and/or liquefaction (Fig. 1 *G* and *H*). Microscopically, both experimental groups also presented with extensive necrotic granulomas. *M. bovis* and *M. orygis* showed comparable bacterial burdens in both the TBLN and MLN, 4-log_10_ higher than the *M. tb* group (Fig. 1 *G* and *H* and *SI Appendix*, Fig. S3 *A* and *B*). Beyond the lymph nodes, a subset of *M. orygis*–infected calves had surface lesions observed in the liver (n = 2/4) but not spleen, though these two calves were bacteriologically positive at both sites (*SI Appendix*, Table S1 and Fig. S4).

### Experimental Murine Infections Recapitulated Differences in Virulence.

**Fig. 2.**
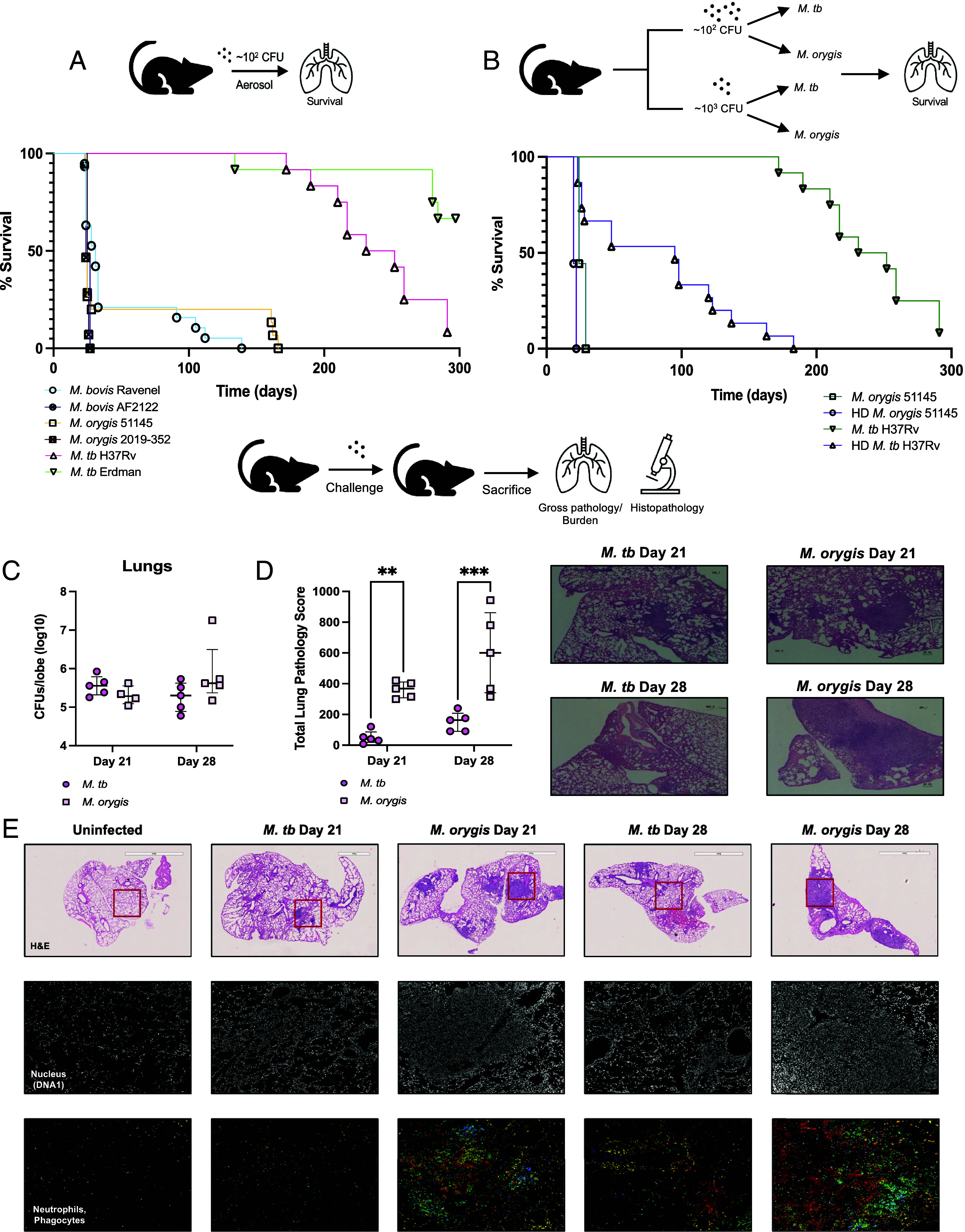
Differential virulence is recapitulated in the murine model. (*A*) Kaplan–Meier survival curves of *M. orygis* 51145*, M. orygis* 2019-352, *M. bovis* Ravenel, *M. bovis* AF2122/97, *M. tb* H37Rv, and *M. tb* Erdman. Mice were exposed to ~200 CFU of various members of *M. bovis* Ravenel*, M. bovis* AF2122/97, *M. orygis* 51145, *M. orygis* 2019-352 (each n = 18) or *M. tb* H37Rv, *M. tb* Erdman (n = 15). Median survival: *M. bovis* Ravenel = 31 d, *M. bovis* AF2122/97= 25 d, *M. orygis* 51145 = 24 d, *M. orygis* 2019-352 = 25 d; *M. tb* H37Rv = 241 d. Median survival could not be determined by experimental endpoint for *M. tb* Erdman. (*B*) Kaplan–Meier survival curve of *M. tb* H37Rv and *M. orygis* 51145 at standard dose compared to high dose (HD) ~1,300 CFU. Standard *M. tb* H37Rv curve generated from independent experiment (n = 12 mice). Median survival of standard *M. orygis* 51145= 24 d (n = 9 mice). Median survival of HD *M. tb* H37Rv= 95 d (n = 15 mice). Median survival of HD *M. orygis=* 20 d (n = 9 mice). (*C*) Lung bacterial burden of *M. tb* H37Rv and *M. orygis* 51145*–*infected groups at day 21 and 28 (n = 5 mice per timepoint per experimental group) (*D*) *Left*: total lung pathology score based on semiquantitative score (*SI Appendix*, Table S2). *Right*: associated hematoxylin and eosin (H&E) staining of right accessory lobe of lung at day 21 or day 28 (4x magnification). Featured slides representative of material sent to pathologist for assessment. (*E*) Single cell imaging mass cytometry readouts (SC-IMC). H&E staining of right accessory lobe following *M. tb* or *M. orygis* infection at day 21 or day 28. Uninfected control obtained from age-matched C57BL/6 female mouse on day 21. The red square represents 1 mm^2^ region of interest (ROI) acquired. Images shown are representative of median score per experimental group reported in Fig. 2*D*. Slides prepared sequentially, from the same samples used in H&E staining. Populations of interest: nuclear DNA (DNA1), neutrophils (MPO, Ly6G, Ly6C-Ly6G), macrophages (CD206), and phagocytes (CD68). Population markers and respective colors in *SI Appendix*, Table S3. DNA1: white, MPO: green, Ly6G: blue, Ly6G-Ly6C: pink, CD206: yellow, CD68: red. Images generated with MCD™ Viewer v.1.0.560.6 (post-acquisition pseudocoloring). Statistical testing: Mantel-Cox test for Kaplan–Meier survival curves; 2-way ANOVA for pathology scores. * indicates *P* < 0.05, ** indicates *P* < 0.01, *** indicates *P* < 0.001.

The differential virulence observed following bovine infection was also observed in a murine model. Mice were exposed to multiple strains of either *M. tb, M. bovis,* or *M. orygis* via the aerosol route (~150 to 300 CFU). Notably, the median survival time following challenge with *M. orygis* or *M. bovis* strains was comparable (24 to 31 d), whereas median survival for the *M. tb* groups was greater than 200 d (Fig. 2*A*). We then tested whether this survival difference could be overcome with a higher dose. When infected with ~1,300 CFU of *M. tb*, the median survival was reduced to 95 d but remained almost three times greater than the median survival observed with standard-dose *M. orygis* (Fig. 2*B*). By this comparison, the lethal dose 50 (LD_50_) of the two animal-lineages is an order of magnitude lower than that of *M. tb.*

To determine the cause of this premature death, we assessed bacterial counts and pathology, comparing mice infected with either *M. orygis* or *M. tb* at day 21 and 28. At both time-points, there were no differences in lung bacterial burdens between *M. orygis* and *M. tb* (Fig. 2*C*). Additionally, no differences in bacterial burden could be observed outside the lungs, such as in the spleen or bone marrow (*SI Appendix*, Fig. S5 *A* and *B*). However, blinded examination of H&E-stained slides of the right accessory lobe by a clinical pathologist revealed striking differences as measured by a predetermined histopathology scale (*SI Appendix*, Table S2). In both groups (*M. orygis, M. tb*), the pathology increased from day 21 to 28. At both timepoints, a marked difference between pathogens was recorded; in fact, *M. orygis*–infected mice had higher pathology scores at day 21 than the *M. tb* group at day 28 (Fig. 2*D*). Notably, prominent granulomatous and/or neutrophilic inflammation with varying degrees of necrosis was only observed in *M. orygis*–infected mice.

To further characterize differences in histopathology at these time-points, we used single-cell imaging mass cytometry (SC-IMC) to quantify immune markers following infection compared to uninfected controls (*SI Appendix*, Table S3). ROIs were selected based on pathology seen with H&E staining on days 21 and 28 (Fig. 2*E*). The most notable differences were 1) *M. orygis* recruited more immune cells compared to *M. tb,* as early as day 21, 2) this recruitment was not seen in the *M. tb* group at the same timepoint, and 3) *M. orygis* infection was dominated by neutrophils, macrophages, and myeloperoxidase (MPO)-producing cells (Fig. 2*E* and *SI Appendix*, Fig. S6 *A* and *B*). In the *M. tb* group, markers for B-cells (B220) and dendritic cells (CD11c) at day 28 were the most comparable to *M. orygis* at either timepoint assessed (*SI Appendix*, Fig. S6 *A*, *C*, and *D*).

To address potential ROI selection bias, we also performed immunohistochemistry (IHC) on entire tissue sections using antibodies specific for neutrophils (Ly6G), proinflammatory responses (IL-1 β), and anti-inflammatory responses (IL-10). Consistent with H&E and SC-IMC findings, neutrophils were more numerous in the *M. orygis–*infected groups compared to the *M. tb* groups (*SI Appendix*, Fig. S7*A*). The proinflammatory IL-1 β staining correlated with the pulmonary lesions, while IL-10 staining was minimal across all experimental groups (*SI Appendix*, Fig. S7 *B* and *C*). Combined, median survival, lethal dose, and lung pathology in C57BL/6 mice recapitulated virulence hierarchies observed in the calves.

### Outcome of Murine Infection Depended on Shared and Lineage-Specific Virulence Factors.

**Fig. 3.**
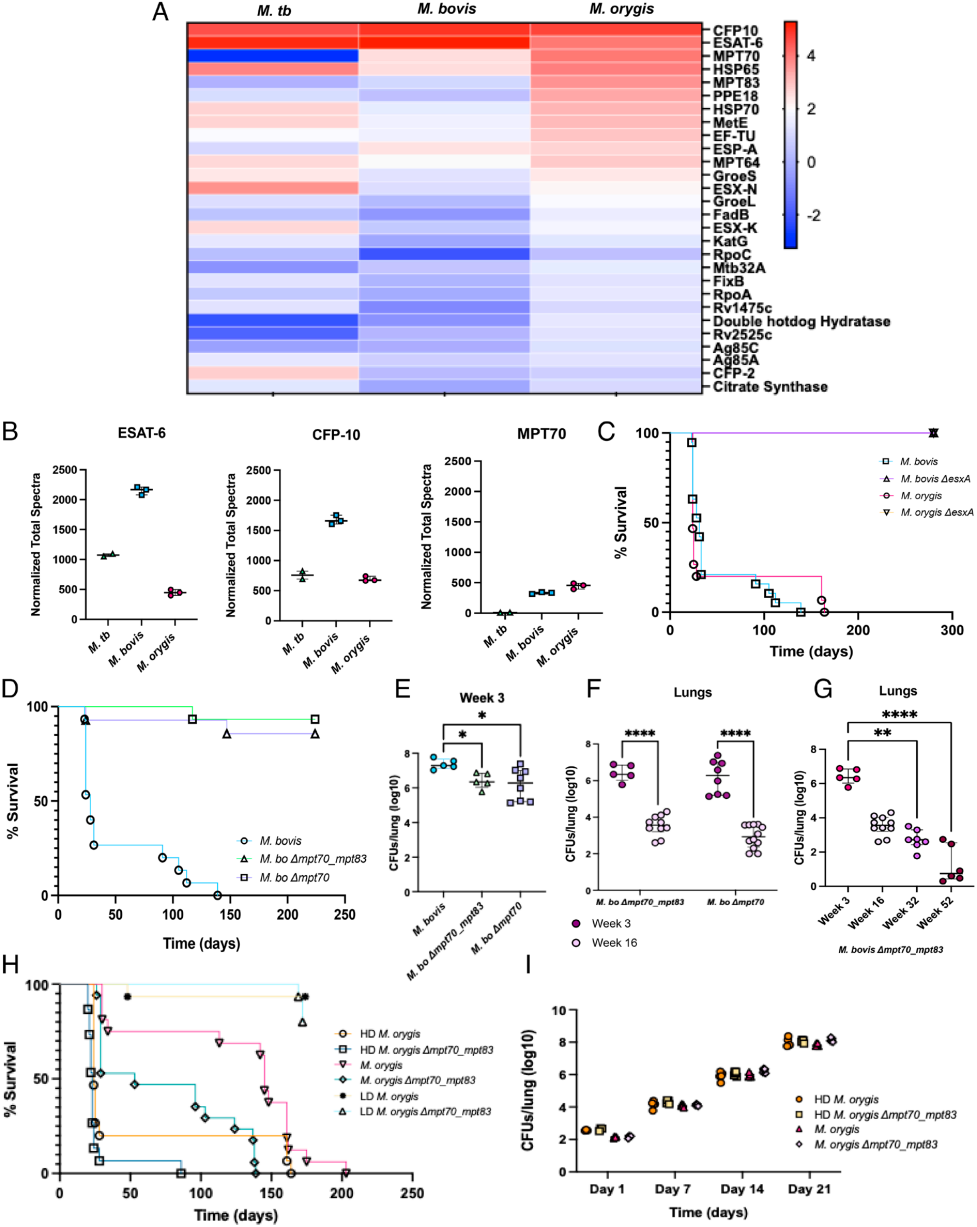
Secretomic comparisons reveal potential drivers of MTBC virulence. (*A*) Comparative heat-map of *M. tb, M. bovis,* and *M. orygis* secretomes (z-score). Each row represents a specific protein; each column represents the average of sample replicates (*M. bovis* n = 3, *M. orygis* n = 3; *M. tb* n = 2). Color intensity indicative of abundance of protein. Heatmap generated using GraphPad Prism v.10.0.3. (*B*) Normalized total spectra counts of ESAT-6, CFP-10, and MPT70 (Scaffold_5.2.1, Proteome Sciences). (*C*) Kaplan–Meier survival curve of two cattle-associated isogenic strains (*M. bovis ΔesxA* and *M. orygis ΔesxA*) compared to their respecitive parental strains, until experimental endpoint (T ~ 300 d, n = 18 mice per group). The *ΔesxA* curves are overlaid. (*D*) Kaplan–Meier survival curve of *M. bovis, M. bovis Δmpt70_mpt83*, and *M. bovis Δmpt70*. Experimental endpoint of *M. bovis Δmpt70_mpt83* and *Δmpt70*= 32 weeks (224 d). Dosing: WT *M. bovis ~*170 CFU, *M. bovis Δmpt70_mpt83 ~*250 CFU, and *M. bovis Δmpt70 ~*300 CFU. (*E*) Lung bacterial burden at week 3. *M. bovis* and *M. bovis Δmpt70_mpt83–*infected groups n = 5 mice/group; *M. bovis Δmpt70* group n = 8 mice (2 experiments). Statistical testing by Kruskal–Wallis test (*F*) Lung burden of *M. bovis Δmpt70_mpt83* and *M. bovis Δmpt70* at 3- and 16-wk. Statistical testing by 2-way ANOVA. (*G*) Lung burden of *M. bovis Δmpt70_mpt83* at week 3, 16, 32, and 52. (*H*) Kaplan–Meier curve of WT or isogenic *(Δmpt70_mpt83*) *M. orygis* at low (~25 CFU), standard (~150 to 200 CFU), or high (~400 CFU) dose. LD = low dose; HD= high dose. (*I*) Lung bacterial burden of *M. orygis* or *M. orygis Δmpt70_mpt83* at days 7, 14, and 21 postinfection (n = 5 mice per timepoint per group). * indicates *P* < 0.05, ** indicates *P* < 0.01, *** indicates *P* < 0.001.

Compared to *M. tb, M. bovis* and *M. orygis* do not contain unique genes (*SI Appendix*, Table S4). To explore the observed differential virulence between the three subspecies, we performed proteomic analyses of *M. tb, M. bovis,* and *M. orygis* culture filtrates. The dominant secreted proteins of *M. bovis* and *M. orygis* were ESAT-6, CFP-10, and MPT70. In contrast, for *M. tb*, ESAT-6 and CFP-10 were the most abundant proteins but MPT70 spectra were rare (Fig. 3 *A* and *B*). Notably, ESAT-6, CFP-10 (two canonical virulence factors), and MPT70 (encoded by *Rv2875* in *M. tb*; *Mb2900* in *M. bovis*; *RJtmp_002967* in *M. orygis*) are identical across these organisms at the nucleotide and amino acid level, suggesting that differences in their levels of expression may be important for infection outcome.

To test this hypothesis, we developed deletion strains in both *M. bovis* (Ravenel) and *M. orygis* (51145) (*SI Appendix*, Table S5). First, we generated *esxA* mutants, to disrupt ESAT-6, and indirectly, CFP-10 (13, 14). Following infection, both *M. bovis* and *M. orygis* showed a complete reversal of the mortality phenotype when *esxA* was disrupted, indicating that ESAT-6 is a critical virulence factor for *M. bovis* and *M. orygis* (Fig. 3*C*, survival curves superimposed). MPT70 is expressed as part of the SigK regulon, along with the homologous protein MPT83—also increased in the *M. bovis* and *M. orygis* secretomes (Fig. 3*A*). As such, we generated deletion strains across *Rv2875 (mpt70)* and *Rv2873 (mpt83)*, calling this “*Δmpt70_mpt83*.” Strikingly, like *ΔesxA, mpt70_mpt83* disruption resulted in near-complete reversal of the *M. bovis* mortality phenotype (Fig. 3*D*). These mice showed a ~1 log_10_ reduced bacterial burden compared to WT *M. bovis* as early as week 3 (with limited lung pathology) and a further 5-log_10_ decrease in bacterial burden between weeks 3 and 52, where a subset showed bacterial clearance in the lungs (Fig. 3 *E*–*G* and *SI Appendix*, Fig. S8 *A*–*C*). When only *Rv2875 (mpt70)* was disrupted, these findings were recapitulated, where only 2 out of 45 mice succumbed to infection by week 24 and lung bacterial burdens declined over time (Fig. 3*D*). Histopathology was concordant with these findings (*SI Appendix*, Fig. S8*D*). In contrast, when *M. orygis* was disrupted for these same genes, the lethal phenotype could not be reversed, regardless of the dose given, and no reduction of bacterial burden was observed compared to wildtype *M. orygis* (Fig. 3 *H* and *I*). In fact, at a low dose (~25 CFU), the WT *M. orygis* and *M. orygis Δmpt70_mpt83* showed a comparable increase of bacterial burden in the lung and spleen between weeks 4 and 16, in addition to progressive lung pathology culminating in several mice succumbing to infection by day 170 (Fig. 3*H* and *SI Appendix*, Fig. S9 *A*–*D*). This suggests that mortality can be modulated in a dose-dependent manner but that this modulation is MPT70/MPT83-independent in *M. orygis*.

### *M. orygis* Mortality Is Modulated By Immunological History.

**Fig. 4.**
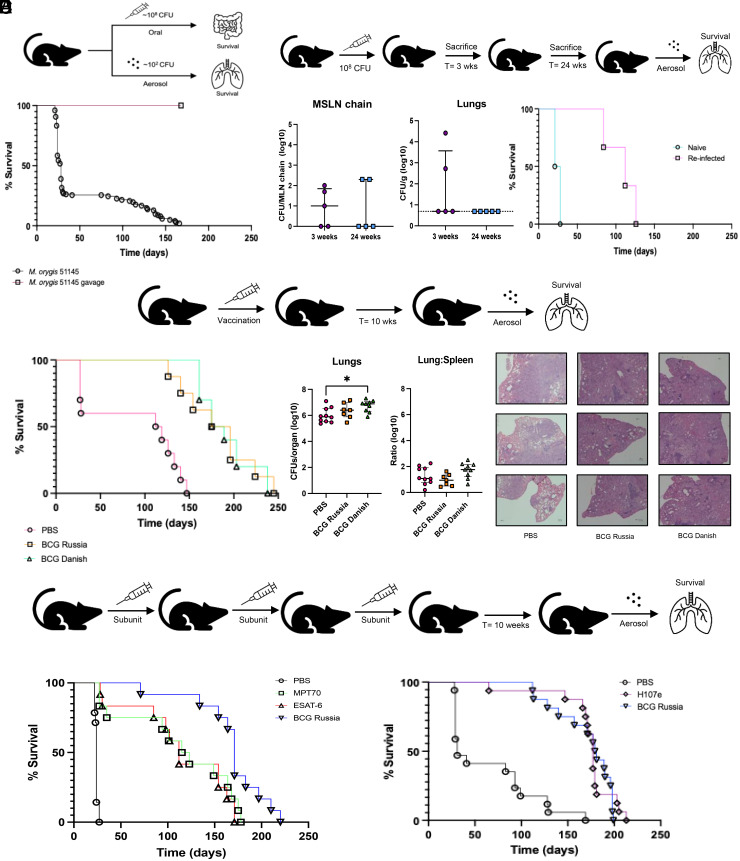
Host immunological status modulates *M. orygis* infection. (*A*) Mice were challenged with WT *M. orygis* either orally (gavage) or via the aerosol route. Dose of oral infection ~10^8^ CFU; n = 15 mice infected. Aerosol survival curve is pooled data from 11 independent experiments at ~140 to 400 CFU (n = 149 mice). Median survival of *M. orygis* oral infection could not be reached by experimental endpoint; median survival of *M. orygis* via the aerosol route= 24 d. (*B*) Following oral infection described in Fig. 4*A*, infection status of the animal was assessed in the me senteric lymph nodes (MSLN chain) and the lungs (n = 5 mice per timepoint). At week 24, remaining orally infected mice were rechallenged with ~200 CFU of aerosolized *M. orygis* (n = 5) alongside age- and sex-matched naive controls (n = 5). Median survival of naive *M. orygis* mice= 24.5 d; median survival of M. orygis rechallenge= 112 d. (*C*) Kaplan–Meier survival curve of PBS sham-vaccinated, BCG Russia vaccinated, or BCG Danish vaccinated mice (n = 10 mice per group). Median survival: PBS = 115.5 d (4/10 mice succumbed by 28 d), BCG Russia= 185.5 d, BCG Danish= 182 d. A single BCG Russia vaccinated mouse survived until experimental endpoint (n = 252 d). (*D*) *Left*: Lung bacterial burden at compassionate endpoint. *Middle*: lung-to-spleen ratio at compassionate endpoints. *Right*: H&E staining of right accessory lobe of mouse at compassionate endpoint (4x magnification). (*E*) Kaplan–Meier survival curve of PBS sham-vaccinated, BCG Russia vaccinated, MPT70 vaccinated, and ESAT-6 vaccinated mice (n = 15 mice per group) following aerosol challenge. Median survival of MPT70 and ESAT-6 vaccinated mice = 119 d and 112 d respectively. Median survival of PBS-sham vaccinated mice = 24 d; BCG Russia vaccinated mice = 171 d. (*F*) Kaplan–Meier survival curve of PBS sham-vaccinated, BCG Russia vaccinated, or H017e-vaccinated mice (n = 15 mice per group) following aerosol challenge. Median survival of PBS-sham vaccinated mice= 31 d; median survival of BCG Russia vaccinated mice= 180 d. Median survival of H107e-vaccinated mice = 177 d.

Animal-adapted strains are often associated with extrapulmonary disease, suggesting the possibility of a noninhalational route of infection (15). We therefore tested whether *M. orygis* could cause disease via gastrointestinal infection (Fig. 4*A*).

Despite an inoculum one million times higher than the aerosol dose (10^8^ CFU versus 10^2^), *M. orygis* gavage resulted in a mild, nonlethal infection, with low bacterial burden in only some of the intestines and/or mesenteric lymph nodes, and limited migration to the lung (Fig. 4*B* and *SI Appendix*, Fig. S10*A*). We then tested whether this controlled infection conferred protection against a subsequent aerosol challenge. Compared to an age-matched group of naive mice, previous gavage completely prevented mortality in the first 28 d, shifting the median survival from 24.5 to112 d (Fig. 4*B*). Using bacteria with differential antibiotic susceptibility, we determined that at the time of euthanasia, the majority of lung bacteria were from the aerosol challenge, indicating the prior intestinal infection had modulated the course of disease but not blocked the secondary infection (*SI Appendix*, Fig. S10 *B* and *C*).

Given the effect of prior infection, we asked whether BCG immunization could recapitulate or exceed this protection. We tested two BCG strains (BCG Russia, BCG Danish) as BCG Russia constitutively produces MPT70 and MPT83, whereas BCG Danish does not (16). Following *M. orygis* challenge, both BCG strains prolonged survival to ~180 d, 160 d longer than the PBS group, with no difference observed between BCG strains (Fig. 4*C*). Additionally, though BCG has been proposed to better prevent extrapulmonary TB, in these infections, the lung:spleen ratio was unaffected by vaccination (Fig. 4*D*) (17).

Finally, we asked whether insights from bacteriologic comparisons might inform subunit vaccination. Previously, a subunit vaccine containing ESAT-6 alone was shown to confer greater protection than MPT70 alone against *M. tb* challenge(18). However, following *M. orygis* infection, ESAT-6 and MPT70 vaccination both prolonged survival to a comparable degree, from 24 d to 112 and 119 d, respectively. This is significantly longer than the sham-vaccinated group, but not as long as BCG (171 d) (Fig. 4*E*). Expanding on these observations, we then tested whether H107e (CAF®), a multisubunit vaccine containing 8-antigens (including ESAT-6, MPT70, and MPT83) would confer greater protection (19). When exposed to the same challenge strain, H107e vaccination achieved the same prolongation of survival as observed for BCG (median survivals = 177 d and 180 d respectively) (Fig. 4*F*).

## Discussion

The idea that different lineages of *M. tb* (*sensu stricto*) present with different levels of virulence is gaining increased recognition(20). Our data reinforce and build upon this notion, by demonstrating dramatically different levels of virulence when comparing organisms across a broader range of the MTBC. Traditionally, bacterial virulence is defined as the manifestation and severity of disease pathology, including death, in a susceptible host (21). In this study, we define virulence accordingly and show that, compared to *M. tb, M. bovis* and *M. orygis* manifest greater virulence, across different metrics, in two different hosts. Our gene disruption data implicate MPT70 as a noncanonical virulence factor that, in part, explains the virulence of *M. bovis*. In the case of *M. orygis*, our immunization data with the MPT70 single subunit vaccine and the H107e vaccine (containing MPT70) indirectly support a role for this protein in the outcome of *M. orygis* infection. However, disruption of the same gene in *M. orygis* did not result in attenuation of virulence, whether measured by bacterial burden or survival. These findings suggest the possibility that *M. orygis* has additional virulence determinants that remain to be discovered, such as categorical or quantitative differences in PE/PPE proteins, mycobacterial lipids, or combinations thereof.

These comparative infection studies not only highlight quantitative differences in pathology between *M. tb* and *M. orygis*, based on scoring, bacterial burden, and time to death, but also qualitative differences in the host response. It is common knowledge that human TB and zoonotic TB are different, despite the causative organisms sharing a conserved genome. Neutrophilic lesions, advanced necrosis, and poorly controlled hyperinflammatory infection have previously been described following natural and experimental *M. bovis* infections in a wide number of hosts (i.e., cattle, elk, deer, goats, and domestic pigs) as well as in postmortem examinations of wild ungulates which naturally succumbed to *M. orygis* infection (3, 22–27). Whether this neutrophilic pathology is due to a distinct type of host response to these pathogens, or rather is the consequence of an infection that overwhelms classical host responses, requires further investigation.

While *M. tb* primarily causes disease in humans, with occasional spillover events, epidemiological investigations show that *M. bovis* and *M. orygis* are maintained in a broad range of species. This indicates that these organisms have a capacity for both intraspecies and interspecies transmission (15, 28). While one well-documented route of transmission from bovine hosts to humans is by the intestinal tract (often through milk consumption), our results suggest that this route is less efficient than aerosol infection, as evidenced by a million-fold higher dose resulting in less disease (29). These data suggest that successful cross-species transmission requires either exposure to a high pathogen burden or alternative routes of transmission.

In the mid-20^th^ century, an inverse relationship was noted between human TB mortality and the incidence of bovine TB in England, suggesting that natural exposure to *M. bovis* through milk might confer protection against human TB (30). Our findings are consistent with this hypothesis and raise the question of what may occur in the future if control measures focus on human TB or zoonotic TB alone. To limit livestock disease and reduce spread to spillover reservoirs, vaccination is an attractive option. However, BCG vaccines have not been implemented due to cross-reactivity with conventional diagnostic tests (31). Our studies highlight the potential for a rapid vaccine testing protocol based on host pathology, rather than bacterial counts, and demonstrate that subunit vaccination can protect as well as BCG. Given the proteomic differences between these organisms, our data also suggest that an opportunity in TB vaccine research is to develop distinct human and zoonotic TB vaccines, based on lineage-associated antigenic profiles.

Our study has several limitations. We used only two zoonotic lineages (2 strains each) and did not test other members of the MTBC that have also been associated with disease in humans, such as *M. africanum*, *M. pinnipedii*, and *M. caprae* (1). Likewise, the *M. tb* strains used were both from lineage 4.Though the results we obtained were consistent with those described for multiple *M. tb* lineages following aerosol infection of C57BL/6 mice (32), head-to-head comparisons of different *M. tb* lineages with *M. orygis* and *M. bovis* may contribute further insights into MTBC virulence. We studied just two hosts (*Bos taurus* calves and C57BL/6 mice) out of the many known to harbor *M. bovis* and *M. orygis.* It is anticipated that a greater understanding of how these organisms cause disease will emerge from data on other hosts, such as guinea pigs or zebu (*Bos indicus*) cattle. Finally, an additional limitation is the size of the animals we studied, as we cannot assess the “true” CFU burden of the entire calf lung, lymph nodes, spleen, or liver.

Our comparative analyses reveal that studying any single member of the MTBC in isolation provides an incomplete understanding of TB pathogenesis. The enhanced virulence of *M. bovis* and *M. orygis* not only highlights their significance as pathogens but also offers unique insights into mycobacterial pathogenesis. The demonstrated ability of these organisms to maintain transmission cycles across multiple host species underscores their importance for both animal and human health, particularly in regions lacking systematic animal TB control programs. The emergence of *M. orygis* as a significant public health concern, despite its relatively recent recognition as a distinct MTBC member, emphasizes the critical need to understand the mechanisms that enable such successful multihost pathogens.

## Materials and Methods

Detailed methods available in *SI Appendix*, *Supplemental Methods*.

### Bacterial Strains.

Natural and engineered mutants used are listed in *SI Appendix*, Table S5.

### Phthiocerol Dimycocerosates (PDIM) Analysis.

The presence of PDIM in our bacterial strains was confirmed prior to all animal infection using the protocol previously described by Reed et al. (33).

### Bovine (*Bos taurus*) Experiments.

Holstein calves (age 6 to 7 wk, post weaning) were infected with 10^4^ colony-forming units (CFU)/calf of *M. tb* H37Rv, *M. bovis* AF2122/97, or *M. orygis* 51145 via the aerosol route and monitored until experimental endpoint (15 wk postinfection). At necropsy, whole lungs, spleen, liver, and lymph nodes (tracheobronchial, TBLN; mediastinal, MLN) were extracted for visual inspection and/or palpation. Parameters of lesion scoring are described in *SI Appendix*, Table S6.^5^ Spleen, liver, TBLNs, and MLNs were visually assessed for the presence or absence of lesions but not scored. Total body weight at endpoint was recorded for each calf (*SI Appendix*, Fig. S3). The calf trial was conducted in the large-animal biosafety level 3 containment rooms at VIDO and was in accordance with guidelines of the Canadian Council on Animal Care (CCAC) and approved by the Animal Research Ethics Board of the University of Saskatchewan (Animal Use Protocol # 20220089).

### Murine (*Mus musculus*) Experiments.

All mouse experiments used 8 to 12-wk-old female or male C57BL/6 mice from Jackson Laboratories unless otherwise specified. For aerosol infection, mice were exposed to either low dose (~25 CFU), standard dose (~150 to 300 CFU), or high dose (~1,300 to 1,400 CFU) *M. tb* H37Rv, *M. bovis* Ravenel, *M. orygis* 51145, or engineered variants. For gastrointestinal infection, we generated streptomycin-resistant *M. orygis* and infected orally after streptomycin pretreatment. All protocols involving mice followed the guidelines of the Canadian Council on Animal Care (CCAC) and mice were euthanized according to the approved procedure by the Animal Ethics Committee of the RI-MUHC animal resource division (AUP # MUHC-7656). Criteria for compassionate endpoint outlined in *SI Appendix*, Table S7.

### Histopathology.

Bovine and murine tissues were fixed in formalin for staining with H&E and assessed by a reader blinded to pathogen and experimental timepoint. Additional modalities for examination of tissue include SC-IMC and IHC as described in supplemental methods (34).

### Bacterial Burden.

Tissue was extracted from each sample and raw bacterial counts were enumerated by plate dilution using standard mycobacterial culturing. CFU are presented as either the total number of bacteria (per organ) or bacteria per gram of tissue.

### Protein Extraction.

Culture filtrate from liquid mycobacterial cultures was subject to mass spectrometry, and raw data were compared to protein sequence database of the imputed common ancestor (MTBC_0_) to generate normalized total spectra counts (35).

### Engineered Bacterial Mutants.

Isogenic *M. bovis* and *M. orygis* strains were generated using ORBIT recombineering; streptomycin-resistant (Strep-R) *M. orygis* was generated using the pNIT:ET system, as detailed in *SI Appendix*, *Supplemental Methods* (36, 37).

### Immunization.

Mice were injected subcutaneously with either PBS, the indicated BCG strain, or the specified vaccine. 10 wk postvaccination, mice were challenged with aerosolized *M. orygis* as described above.

### Statistical Analyses.

Survival curves were compared using the Kaplan–Meier (nonparametric) method. Comparison of multiple groups at specific timepoints was performed using Kruskal–Wallis test (Dunn’s multiple comparison test, nonparametric). Comparison of multiple groups over multiple timepoints was performed using an ordinary 2-way ANOVA with Sidak’s multiple comparisons test. Analyses were performed using GraphPad Prism (v10.0.3), and statistical significance was defined as *P* < 0.05.

## Supplementary Material

Appendix 01 (PDF)

Dataset S01 (XLSX)

Dataset S02 (XLSX)

Dataset S03 (XLSX)

## Data Availability

Whole genome sequencing is available on NCBI under BioProject PRJNA1141970 (38). Raw values and associated Z-scores for proteomic and SC-IMC readouts are available as *SI Appendix*. All other data are included in the manuscript and/or supporting information.
