# Supplementary material for: Virulence hierarchies within the Mycobacterium tuberculosis complex
Source: Proc Natl Acad Sci U S A. 2025 Oct 16;122(42):e2507104122. doi: 10.1073/pnas.2507104122 (PMC12557539; doi:10.1073/pnas.2507104122)
Supplement: Supplementary file 1 — Appendix 01 (PDF) [file pnas.2507104122.sapp.pdf]

PNAS

Supporting Information for

Virulence hierarchies within the Mycobacterium tuberculosis complex

Short title: Differential virulence of the MTBC

Sarah N. Danchuk[1,2,3], Shannon C. Duffy[4], Jaryd Sullivan[5], Syed Beenish Rufai[6,7], Fiona A. McIntosh[2,3], Andréanne Lupien[1,2,3,8], Luke B. Harrison[2,8], Hojjat Ghasemi Goojani[2,3], Lorne Taylor[2], Yuhong Wei[9], Philippe Joubert[10], Rasmus Mortensen[11], Jeffrey M. Chen[12], Nirajan Niroula[12], Robin Stevens[12], Carla Norleen[12], Vivek Kapur[13], Marcel A. Behr*[1,2,3,8]

1. Department of Microbiology and Immunology, McGill University, Montreal, QC, Canada
2. Research Institute of the McGill University Health Centre, Montreal, QC, Canada
3. McGill International Tuberculosis Centre, Montreal, QC, Canada
4. Department of Epidemiology of Microbial Diseases, Yale School of Public Health, New Haven, CT, USA
5. Department of Molecular Biology and Centre for Computational and Integrative Biology, Massachusetts General Hospital, Boston, MA, USA
6. Department of Neuroscience, University of Lethbridge, Alberta, Canada
7. Department of Biochemistry and Medical Genetics, University of Manitoba, Winnipeg, Canada
8. Department of Medicine, McGill University Montreal, Montreal, QC, Canada
9. Goodman Cancer Institute, McGill University, Montreal, QC, Canada
10. Institut Universitaire de Cardiologie et de Pneumologie de Québec-Laval University, Quebec City, QC, Canada
11. Center for Vaccine Research, Department of Infectious Disease Immunology, Statens Serum Institut, Copenhagen, Denmark
12. Vaccine and Infectious Disease Organization (VIDO), University of Saskatchewan, Saskatoon, SK, Canada
13. Department of Animal Science and the Huck Institutes of the Life Sciences, The Pennsylvania State University, University Park, PA, USA

Correspondence to: Prof Marcel A. Behr, Research Institute of the McGill University Health Centre, Montreal, QC, H4A 3J1, Canada, marcel.behr@mcgill.ca

This PDF file includes:

Supplemental Methods
Supplemental Results
S Figures 1 to 12
S Tables 1 to 8
Legends for S Data 1 to 3
SI References

Other supporting materials for this manuscript include the following:

S Data 1 to 3

Supplemental Methods

Bacterial strains: All wildtype and parental strains used in this experiment are listed in S Table 5. Streptomycin-resistant *M. orygis* was generated using the pNIT:ET method, as previously described.(1) Strains containing targeted gene-deletions were generated using the "oligo-mediated recombineering followed by Bxb1 integrase targeting" (ORBIT) system as described by Murphy et al. with modifications.(2) Cultures were recovered for 6 days in 7H9 complete media (defined as 7H9 + 0.1% Tween-80 + 0.2% glycerol + 10% ADC (albumin, dextrose, and catalase, Beckton Dickinson BBL Middlebrook)) following electroporation and incubated up to 6 weeks at 37°C. Colonies were screened using conventional PCR; gene deletions were confirmed with Sanger sequencing (Genome Quebec) and qRT-PCR. Subsequently, deletion strains were subject to whole genome sequencing using Illumina NovaSeq 6000 (Genome Quebec) to confirm the fidelity of the gene deletions (available on NCBI under BioProject PRJNA1141970).

Aerosol infection with pre-defined time-points: Inoculum was prepared as described above. 13 mice were exposed to either *M. orygis* 51145 or *M. tb* H37Rv via the aerosol route at a standard dose of ~150-300 CFU. Three mice per group were sacrificed to determine initial dose, as described above. The remaining mice were sacrificed at day 21 and 28 (five mice per experimental group per time point). Both lungs and spleen were collected for downstream processing and accessory lobes of infected mice were extracted for histological assessment (H&E or ZN staining where specified). Slides of the right accessory lobe were reviewed, blinded to experimental group. A semiquantitative scale (S Table 2) was used to score bronchial/endobronchial, peribronchial, perivascular, interstitial, pleural and intra alveolar inflammation, capillary vascular congestion, and pulmonary edema. Additionally, the tissue quality, percent of lung area affected, and the distribution of lesions were assessed in the consideration of these scores (S Table 2). Single-cell imaging and mass cytometry was used to further delineate immune populations at day 21 and 28 as previously described.(3) Briefly, formalin-fixed paraffin embedded (FFPE) slides underwent deparaffinization and heat-mediated antigen retrieval using the Ventana Discovery Ultra auto-stainer platform (Roche Diagnostics) per manufacturer's instructions. Slides were incubated at 70 °C in pre-formulated EZ Prep solution (Roche Diagnostics), followed by incubation at 95 °C in pre-formulated Cell Conditioning 1 solution (Roche Diagnostics). Subsequently, slides were rinsed in 1× PBS and incubated for 45 min at room temperature in Dako Serum-free Protein Block solution (Agilent). An antibody cocktail containing metal-conjugated antibodies was prepared in Dako Antibody Diluent at optimized dilutions. Slides were stained with primary antibodies at 4 °C overnight and subsequently washed with 0.2% Triton X-100 and 1× PBS. A secondary antibody cocktail containing metal-conjugated anti-biotin was prepared in Dako Antibody Diluent at the optimized dilution. Slides were incubated with anti-biotin for 1 hour at room temperature and washed with 0.2% Triton X-100 and 1× PBS. Slides were counterstained with Cell-ID Intercalator-Ir (Fluidigm) diluted at 1:400 in 1× PBS for 30 min at room temperature, rinsed for 5 min with distilled water, and air-dried prior to IMC acquisition. IMC acquisition was performed using the Hyperion™ Imaging System (Fluidigm). Post-acquisition data was visualized using MCD™ Viewer v.1.0.560.6. Markers

described in S Table 3. IHC staining was performed with Ly-6G recombinant rabbit monoclonal antibody by the Goodman Cancer Research Histology Core of McGill University per established methods.

Aerosol challenge in a bovine model: Aerosol challenge was performed in collaboration with the University of Saskatchewan Vaccine and Infectious Disease Organization (VIDO) in an agricultural biosafety containment level 3 (CL3) facility. Bacteria were resuspended in 4 mL of saline solution and nebulized. Fifteen *Bos taurus* calves (six-to-seven weeks, post-weaned) were randomized into *M. orygis* 51145, *M. bovis* AF2122, and *M. tb* H37Rv infection groups (five per group) and challenged with ~10^4 CFU/calf via the aerosol route. Following infection, calves were monitored by the Animal Services Department at VIDO through both physical examination and CCTV footage. Inoculum from each group was plated on 7H10 + OADC agar to determine initial challenge material (100 μL).

Gross pathology: Fifteen weeks (~105 days) post infection, calves were euthanized in three randomized batches. At necropsy, whole lungs, spleen, liver, and lymph nodes (tracheobronchial, TBLN; mediastinal, MLN) were extracted. Individual lobes of lungs were visually inspected and palpated to determine a final lesion score (0-5 per lung, total pathology score= 40). Parameters of lesion scoring are described in S Table 6 (4). A score of 0 indicates no gross lesions seen and/or palpated whereas a score of 5 indicates numerous coalescing lesions of varying sizes observed (S Table 6)(4).

Histopathology: 3 x 3 x 3 cm pieces of tissue were allocated for histopathology (left or right cranial, mid, and caudal lobes of lung; TBLN and MLN). Tissue was stored in 10% neutral buffered formalin and processed by the Goodman Cancer Research Centre Histology Core (McGill University) using standard paraffin embedding protocols. 4 μM slices were cut and transferred to slides to be H&E or ZN stained as indicated.

CFU enumeration: Tissue was extracted from each lobe of lung (both right and left cranial lobes, mid lobes, and caudal lobes), TBLN, MLN, spleen, and liver using a sterile biopsy punch (~1 g) and plated on 7H10 PANTA OADC plates. For CFU interpretation, any raw bacterial counts under 10 were excluded from CFU quantification in lung, MLN, and TBLN. Due to the size of calf, burden in all organs processed is calculated as CFU/g (log_{10}). Lung bacterial burden is presented as the sum of left and right caudal, middle, and cranial lobes (CFU/g).

Preparation of aerosol stocks for murine infection: Mycobacterial strains were grown in 20 mL of 7H9 complete media to mid-log phase (OD_{600}= ~0.8-1) and passaged twice. Following the second passage, cultures were centrifuged at 500 rpm for 5 minutes. Supernatant transferred to new 50 mL conical and centrifuged at 3980 rpm for 10 minutes. The pellet was resuspended in 1 mL 7H9 complete media and passed through a 25g syringe 5 times to remove any remaining clumps. Following this, 19 mL of 7H9

complete media was added to a total of 20 mL. 100% sterile glycerol was added to a final concentration of 10% and frozen at -80°C. Aerosol stocks for all animal experiments were prepared as described above unless otherwise specified.

MTBC survival experiments: Frozen aerosol stocks were thawed and diluted in PBS supplemented with 0.05% Tween-80. 18 C57BL/6 female mice were exposed to a continuous stream of aerosolized inoculum for 15 minutes (CH Technologies Nose-Only Inhalation exposure system) unless otherwise specified. Following infection, inoculum was plated (day 0) on 7H10 agar plates containing polymyxin B, amphotericin B, nalidixic acid, trimethoprim, azlocillin (PANTA, Beckton Dickinson) and supplemented with oleic acid, albumin, dextrose, and catalase (OADC, Beckton Dickinson BBL Middlebrook). Three mice per group were sacrificed at either day 1 or day 3 (as specified) to determine initial infectious dose received by mice. Lungs were extracted, homogenized in 2 mL of 7H9 complete media and plated on 7H10 PANTA + OADC. Lungs were homogenized using the Omni Tissue Homogeniser and Hard Tissue Omni Tip Plastic Homogenising Probes (Omni International). The remaining mice were monitored by animal health technicians until compassionate (clinical) or experimental endpoint at which a necropsy was performed on the mouse. Compassionate endpoints were defined as mice which have lost > 20% of weight compared to start of infection, hunched posture, inactive or decreased activity, dehydration, and/or having rapid breathing (S Table 7). At necropsy, the right accessory lobe of lung was extracted for downstream hematoxylin and eosin (H&E) or Ziehl-Neelsen (ZN) histological staining where indicated (McGill University Goodman Cancer Research Centre Histology Core). The remaining lungs, spleen, and liver were harvested and homogenized in 1 mL 7H9 complete media. Samples were serially diluted and plated on 7H10 PANTA + OADC to determine bacterial burden after 4-6 weeks of incubation at 37°C. This protocol was used for all survival experiments with the exception of *in vivo M. bovis* studies in which inocula and homogenized organs were plated on 7H11 + PANTA + OADC + glycerol plates. Comparison of 2 groups at a single timepoint was performed using Mann-Whitney U test.

M. orygis 51145 whole genome sequencing: The *M. orygis* 51145 genome sequence was corrected using Illumina HiSeq. Raw Illumina reads were screened using FastQC v0.11.9 and quality score of >= 20 was retained. The output of contigs was assembled using the SPAdes genome assembler (v3.9.0) using the default *k-mer* size, annotated using Prokaryotic genome annotation pipeline (PGAP) and submitted in sequence read archive (SRA) under accession number (SRR16643349). Illumina fastq reads were mapped onto the annotated Pacbio sequence and variants were called using Geneious Software v.2021.1. The list of variants extracted from Illumina sequenced Orygis 51145 genome in comparison with Pacbio Sequenced Orygis 51145 genome is given in S Table 4 and 8. The revised *Mycobacterium orygis* 51145 genome was deposited in GenBank under the accession number CP063804.2.

Genome comparisons: The updated genome of *M. orygis* 51145 was compared against those of H37Rv and *M. bovis* Ravenel. For H37Rv, comparisons were made with the NCBI GenBank (GCA_000195955.2) and RefSeq (GCF_000195955.2) genome annotations, which have been extensively curated, as well as the updated H37Rv-1 assembly and PGAP annotation published by Chitale et al.(5) For *M. bovis* Ravenel, the available GenBank (GCA_018305025.1) and RefSeq (GCF_018305025.1) assemblies and PGAP annotations were used. Genomic feature counts were extracted from the annotations manually. The number of PE and PPE family genes was identified by both manual review of genome annotations and by using hmmsearch v3.4 on the proteomes predicted by each annotation (search profile PF00934 for PE proteins and PF00823 for PPE proteins)(6). The hmmsearch was conducted on both the published annotations and on a uniform re-annotation of each respective genome using prokka v1.14.6(7).

Mycobacterial protein extraction: Cultures of *M. tb* H37Rv, *M. bovis* Ravenel and *M. orygis* 51145 were grown in 10 mL 7H9 complete media to mid-log phase (OD_{600}= 0.8-1) and passaged in 30 mL of Sauton minimal media. Cultures were again grown to an OD_{600} of ~0.8 and passaged an additional two times in 30 mL Sauton media. Following this, cultures were centrifuged at 3980 rpm for 15 minutes. For culture filtrate, supernatants were filtered twice through 0.22 μM filters and concentrated using centrifugal filter units (Amicon Ultra-15 Centrifugal Unit, 3 kDa cutoff) by spinning at 3980 rpm for 2-3 hours to a final volume of 1 mL. Protein concentration was determined using the Qubit Protein BR Assay per manufacturer instruction (Thermo Fisher Scientific, Waltham MA, USA). For each sample, proteins were loaded onto a single stacking gel band to remove lipids, detergents, and salts. The gel band was reduced with DTT, alkylated with iodoacetic acid and digested with trypsin. Extracted peptides were re-solubilized in 0.1% aqueous formic acid and loaded onto a Thermo Acclaim Pepmap (Thermo, 75uM ID X 2cm C18 3uM beads) precolumn and then onto an Acclaim Pepmap Easyspray (Thermo, 75uM X 15cm with 2uM C18 beads) analytical column separation using a Dionex Ultimate 3000 uHPLC at 250 nl/min with a gradient of 2-35% organic (0.1% formic acid in acetonitrile) over 3 hours . Peptides were analyzed using a Thermo Orbitrap Fusion mass spectrometer operating at 120,000 resolution (FWHM in MS1) with HCD sequencing (15,000 resolution) at top speed for all peptides with a charge of 2+ or greater. The raw data were converted into *.mgf format (Mascot generic format) for searching using the Mascot 2.6.2 search engine (Matrix Science) against the protein sequence database of the imputed ancestor of the MTBC (called $MTBC_0$) and a database of common contaminant proteins.(8) The database search results were loaded onto Scaffold Q+ Scaffold_5.2.1 (Proteome Sciences) for statistical treatment and data visualization (AVOVA with Benjamini-Hochberg correction, $p < 0.05$; minimal spectra value= 0.0). (Proteomics and Molecular Analysis Platform, Research Institute of the McGill University Health Centre). Normalized total spectra counts are provided in Supp File 1. Heatmap constructed using secretome Z-scores > 2.58.

Isogenic aerosol infection: Isogenic *M. bovis* and *M. orygis* aerosol survival studies were performed as previously described without modification.

M. bovis Δmpt70_mpt83 aerosol challenge with pre-defined timepoints: Mice were infected via the aerosol route (~200-300 CFU) and randomized to either week 3 (n= 5), week 16 (n= 10), week 32 (n= 7), or week 52 (n= 7), per experimental design. At pre-defined timepoints, mice were sacrificed. The right accessory lobe of lung was extracted for H&E staining; remaining lobes of lung and spleen were processed as described above for CFU enumeration.

M. bovis Δmpt70 aerosol challenge with pre-defined timepoints:
Mice were infected via the aerosol route (~100-300 CFU) and randomized into week 3 (n= 3 or 5, as specified) or week 16 (n= 10) experimental groups (2 independent experiments). At pre-defined timepoints, the right accessory lobe was extracted for H&E staining but only lungs were processed for CFU enumeration.

M. orygis Δmpt70_mpt83 aerosol challenge with pre-defined timepoints: Mice were infected via the aerosol route with ~150-200 CFU (standard dose) or ~400-500 CFU of *M. orygis* or *M. orygis Δmpt70_mpt83* (HD) as previously described and randomized into day 7, day 14, or day 21 timepoints (n=5 mice per timepoint, per group). At pre-defined timepoints, mice were sacrificed as described previously (lungs extracted for CFU enumeration; right accessory lobe allocated for H&E staining).

Low dose *M. orygis* infection: Mice were infected via the aerosol route with ~25 CFU of *M. orygis* or *M. orygis Δmpt70_mpt83* and assessed for clinical disease (n= 18 mice per experimental group). At T= 4- and 16-weeks p.i. randomly selected mice were sacrificed and assessed for bacterial burden and extent of pathology (n= 3 mice at 4-weeks p.i.; 5 mice at 16-weeks p.i.). Remaining mice were monitored until compassionate or experimental endpoint.

M. orygis gavage followed by aerosol challenge: Strep-R *M. orygis* (*M. orygis rpsL* K43R) was grown to an OD_{600} of 0.5 (~7.5×10^7 CFU/mL) in 7H9 complete media. The culture was washed once in PBS + 0.01% Tween-80 and then resuspended in 0.15x the original volume in PBS + 0.01% Tween-80 to generate a 5×10^8 CFU/mL stock. Male and female C57BL/6 mice (n= 15) were infected with 200µL of the prepared strep-R *M. orygis* by oral gavage for a dose of 10^8 CFUs. At weeks 3 and 24, 5 mice were sacrificed and the small intestine (SI), large intestine (LI), mesenteric lymph nodes (MLN), lungs, spleen and liver were harvested. Organs were plated on 7H10 PANTA + OADC, as described above. At week 24, remaining mice were re-challenged with ~150-300 CFU of aerosolized *M. orygis* and monitored as detailed above. At endpoint, a lobe of lung from naïve mice was homogenized in 1 mL 7H9 complete media and plated on 7H10 + OADC + PANTA whereas for mice that had previously been infected with Strep-R *M. orygis* by gavage, lungs were plated on 7H10 + OADC + PANTA with or without streptomycin (50 µg/mL).

Additionally, the intestines were harvested to following established methods to look for evidence of intestinal TB(1).

Vaccination with BCG Russia and BCG Danish: Strains of *M. bovis* BCG Russia and *M. bovis* BCG Danish were grown in 7H9 complete media, passaged twice, and processed to remove any remaining clumps, as described above in the preparation of aerosol stocks. Bacterial cultures were then adjusted to an OD_{600} of 0.1. Following this, BCG Russia or BCG Danish was injected via the subcutaneous route (100 µL; n= 12 mice per group). Additionally, 12 mice were sham vaccinated with 100 µL of phosphate buffered saline (PBS). At 10-weeks post vaccination, all groups were challenged with aerosolized *M. orygis*. Two mice per group were sacrificed at day 3 to determine initial aerosol dose. At compassionate endpoints, lungs and spleen were harvested and processed as described above. Remaining mice were monitored for signs of clinical disease until experimental endpoint at week 36. At experimental or compassionate endpoint, the right accessory lobe was extracted for histopathological studies.

Vaccination with single peptide recombinant antigens: ESAT-6 and MPT70 recombinant antigens were produced with a His-tag at the N-terminal end (MHHHHHH-) and purified in accordance with established methods(9) Codon optimized DNA constructs were inserted into the pJ 411 expression vector (ATUM, Menlo Park, CA, USA) and transformed into *E. coli* BL21 (DE3) (Agilent, DK). In 3L liquid cultures, protein expression was induced with 1 mM isopropyl ß-d-1-thiogalactopyranoside and ESAT-6/MPT70 were purified from inclusion bodies by metal chelate chromatography followed by anion-exchange chromatography. Vaccine formulation were prepared by mixing CAF01 with recombinant ESAT-6 or MPT70 and subcutaneously administered as previously described by Clemmensen et al.(9, 10). In tandem, mice were vaccinated with BCG Russia or sham-vaccinated with PBS, as described above. Ten weeks post vaccination, mice were challenged with standard dose of *M. orygis* 51145 (150-300 CFU) via the aerosol route and monitored for signs of clinical disease until compassionate or experimental endpoint.

Vaccination with fusion peptide recombinant antigens (M72/AS01$_E$ and H107e/CAF01): Mice were vaccinated by intramuscular injection with 3 doses of M72/AS01$_E$ (1 µg/mouse) at 3-week intervals as previously described. In tandem, mice were vaccinated subcutaneously with 3 doses of H017e/CAF01 (2 µg/mouse) at 2-week intervals as described by Dijkman et al.(11) At 10 weeks following the final dose, mice were challenged with *M. orygis* 51145 (standard dose, 150-300 CFUs) via the aerosol route and monitored for signs of clinical disease until compassionate endpoint. As above, vaccination with PBS or BCG Russia were included as infection controls without modification.

Supplemental Results

Differential virulence cannot be attributed to unique genes in *M. bovis* or *M. orygis*

Genome sequencing of *M. orygis* 51145 was previously performed using PacBio single-molecule real-time (SMRT) technology to assemble a single circular chromosome(12). To verify nucleotide reads, we used the Illumina HiSeq platform on the same DNA aliquot, determining that *M. orygis* strain 51145 has a genome size of 4,352,140 bp in length, with discrepancies (missense mutations and deletions) in five genes corrected (S Table 8). We also compared the genomes of *M. tb* H37Rv, *M. bovis* Ravanel, and *M. orygis* 51145 (S Table 4). The three genomes are similar in size (~4.4Mb) and predicted gene content (~4100-4200). Of the three species, *M. tb* has the largest genome, whereas *M. orygis* has the smallest. Chromosome visualized in S Fig 11.

Establishment of the *M. orygis* infection model

We infected C57BL/6 mice via the aerosol route with the intention of assessing bacterial burden and histopathology at days 21, 42, and 84 following infection. Unexpectedly, by day 28, 50% of the *M. orygis* infected group had reached compassionate endpoint resulting in a median survival of 28 days. In contrast, the median survival of *M. tb* infected mice was not reached by experimental endpoint at day 119 (S Fig 12a). Just prior to the wave of mortality, lungs, spleens, and livers had been harvested and processed for bacterial burden, and the right accessory lobe was extracted for histopathology as scheduled on day 21. Samples revealed extensive pulmonary pathology, with little remaining alveoli in *M. orygis* infected mice that succumbed to infection by day 28 (S Fig 12b). Further, bacterial burdens at day 21 were comparable between *M. tb* and *M. orygis* infected mice, with no evidence of increased dissemination of *M. orygis* from the lungs (consistent across 2 independent experiments, S Fig 12c-e). Bacterial burdens in the lung, spleen, and liver were also comparable between day 21 and day 28 in the *M. orygis* group. To standardize this model, we also evaluated whether infection outcome varied with sex. Mortality following *M. orygis* infection was sex independent, with a median survival of 25 days for males and 23.5 days for females (n= 36 mice, 2 independent experiments) (S Fig 12f). Bacterial burden and histopathology at time-matched compassionate endpoint were also comparable (S Fig 12g,h).

As described in this study, we also compared infection outcome of *M. tb* or *M. orygis* at various doses (~200 CFU v. ~1300 CFU) and determined that the median survival of *M. tb* infected mice shifts to a much greater degree at a higher dose, compared to *M. orygis* at that same dose (Fig 2b). This is reflected macroscopically, at the same experimental timepoint. Compared to *M. tb* at either standard or high dose, *M. orygis* infected mice present with visible, aggressive lung pathology as evidenced by numerous, raised lesions. Unsurprisingly, the HD *M. orygis* infected group shows the worst pathology with coalescing lesions comprising virtually all lung space (S Fig 12i).

Supplemental Figures

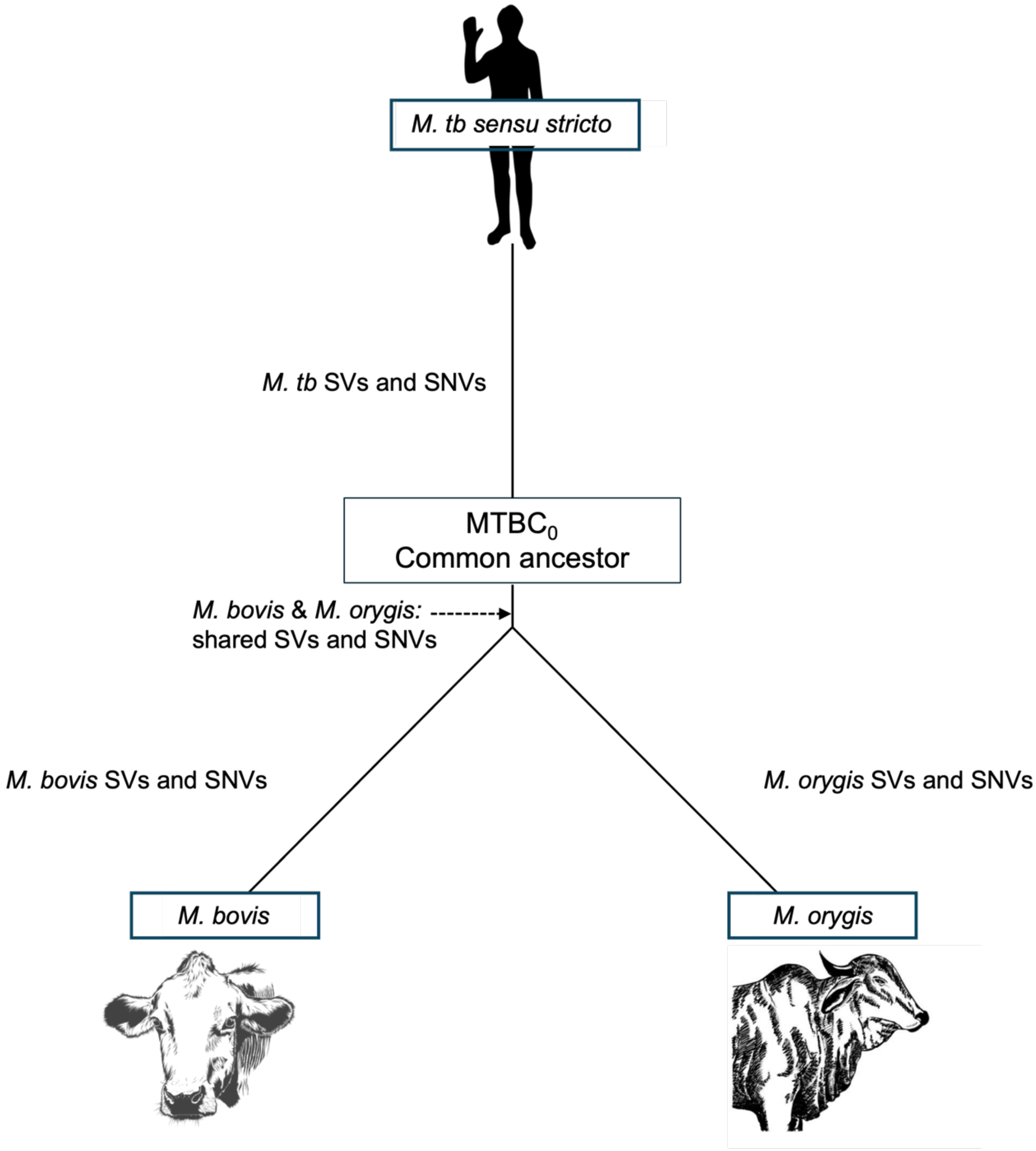

S Figure 1: Evolutionary relationship between *M. tuberculosis, M. bovis,* and *M. orygis*

Since the common ancestor of the *Mycobacterium tuberculosis* complex (MTBC), imputed as $MTBC_0$ evolutionary events have shaped the three organisms under study, namely *M. tuberculosis* (*M. tb*), *M. bovis* and *M. orygis* (Harrison, 2024) (8). These include structural variants (SVs), such as deletions, and single nucleotide variants (SNVs), such as point mutations. *M. tb*-specific deletions include the TbD1 region, absent from the strains of *M. tb* used in this study (Brosch, 2002) (13). *M. bovis* and *M. orygis* have SVs and SNVs in common (e.g., deletion of RD9), but are also distinguished by lineage-specific deletions (including different RD12 deletions) (Mostowy, 2005) and lineage-specific mutations (e.g., distinct *rskA* mutations that independently result in upregulated MPT70 expression) (Saïd-Salim,2006) (14, 15). Phylogenetic scale approximated from whole genome sequence-based phylogeny (Duffy, 2020)(16).

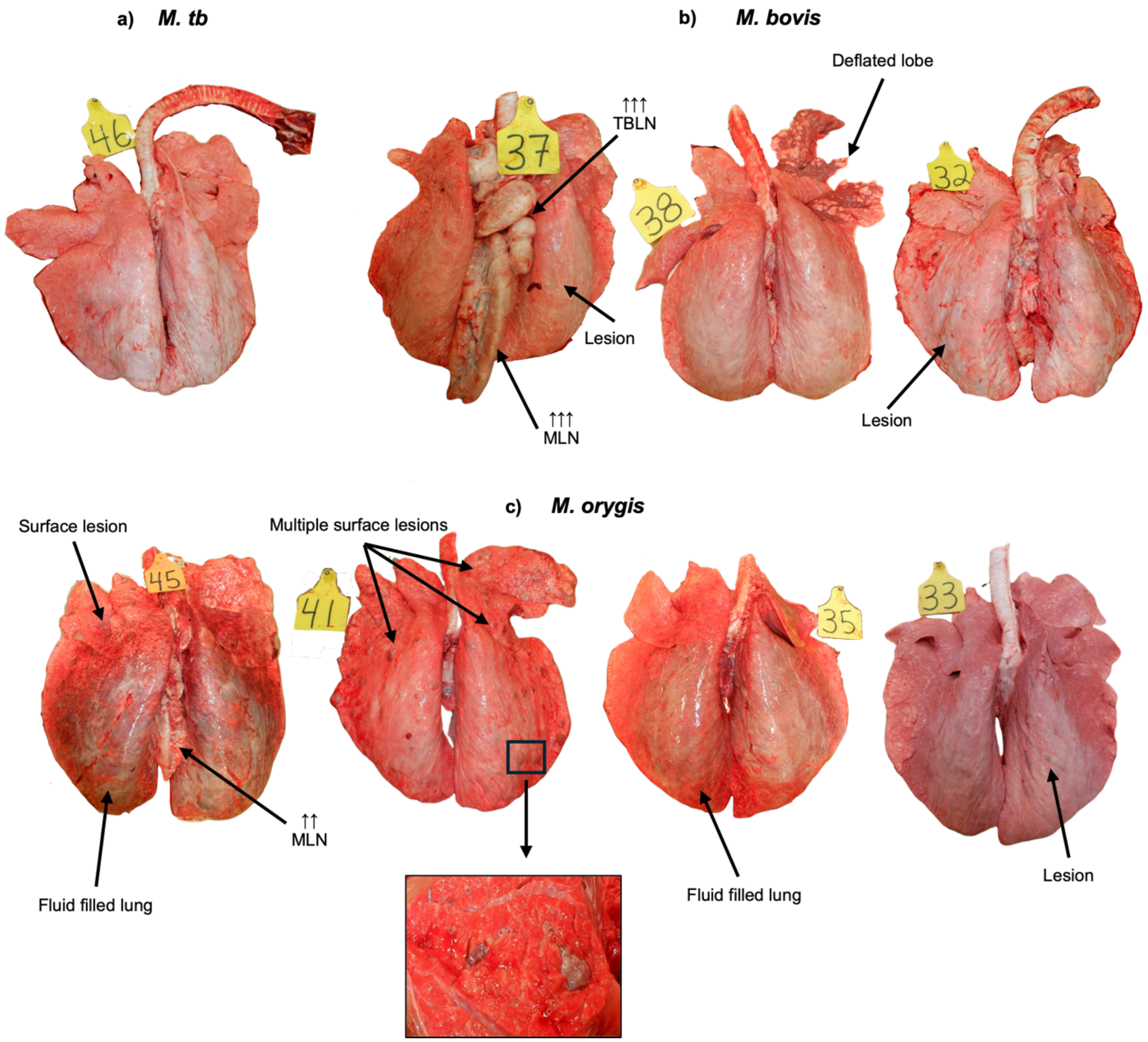

S Figure 2. Comparative gross pathology of MTBC-challenged lungs.

a) Surface pathology of *M. tb* negative control. **b)** Surface pathology of all surviving *M. bovis* infected calves (n= 3). Highest gross pathology score= calf 37, 30/40; lowest gross pathology score= calf 32, 9/40. Notable features highlighted: size of TBLN, MLN, examples of lesions, collapsed lung (unrelated to infection). **c)** Surface pathology of all surviving *M. orygis* infected calves (n= 4). Highest gross pathology score= calf 45, 36/40; lowest gross pathology= calf 33, 12/40. Overall ranking: calf 45 > calf 41 > calf 35 > calf 33. Notable features highlighted: edema, surface lesions. Black box inset: details of surface lesion following transverse slicing of lung. Calf 46 representative of *M. tb* infected cattle.

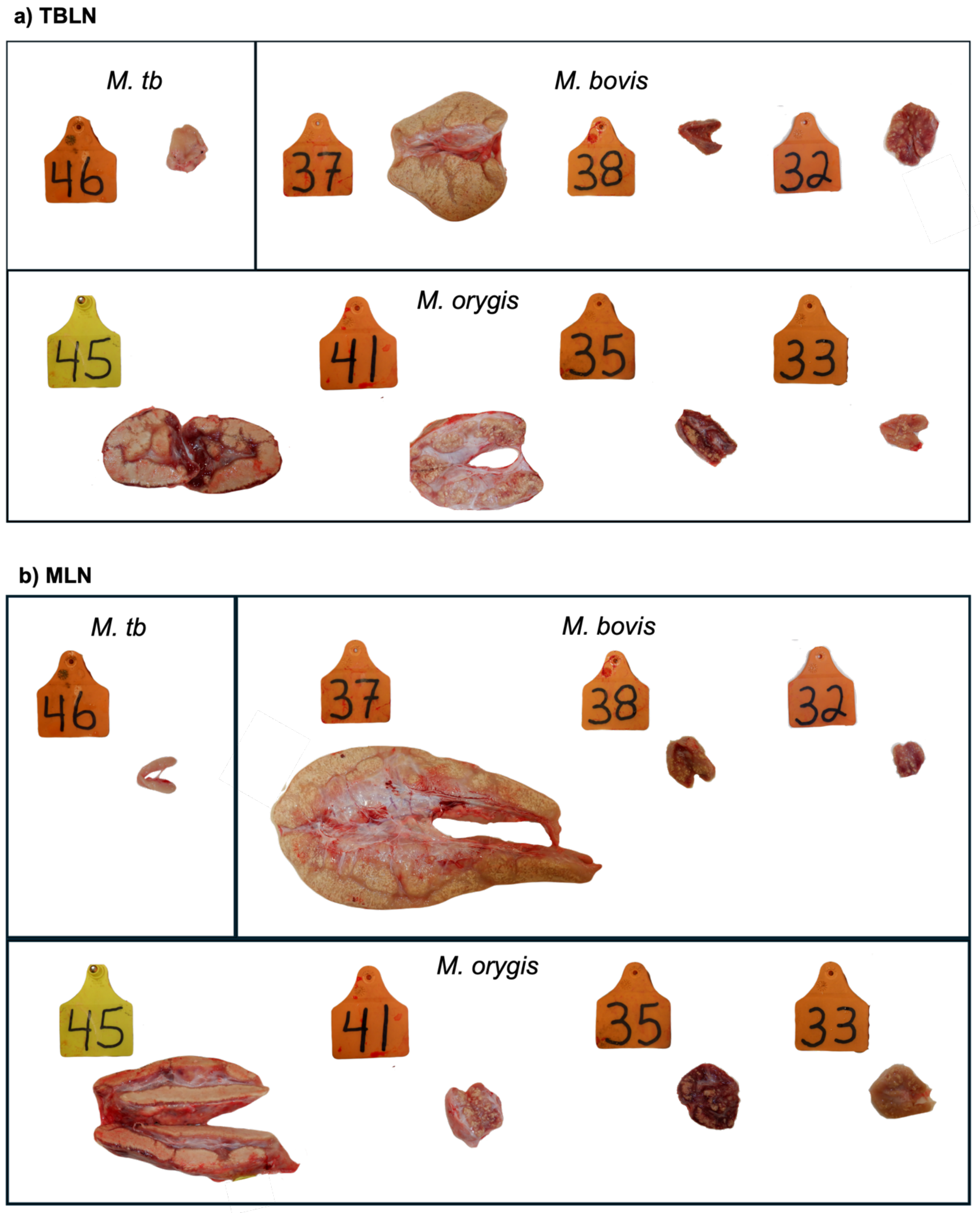

S Figure 3. Comparative gross pathology of MTBC-challenged TBLNs and MLNs.

a) TBLN from *M. tb, M. bovis* and *M. orygis* infected cattle. **b)** MLN from *M. tb, M. bovis,* and *M. orygis* infected cattle. Calf 46 representative of *M. tb* infected cattle.

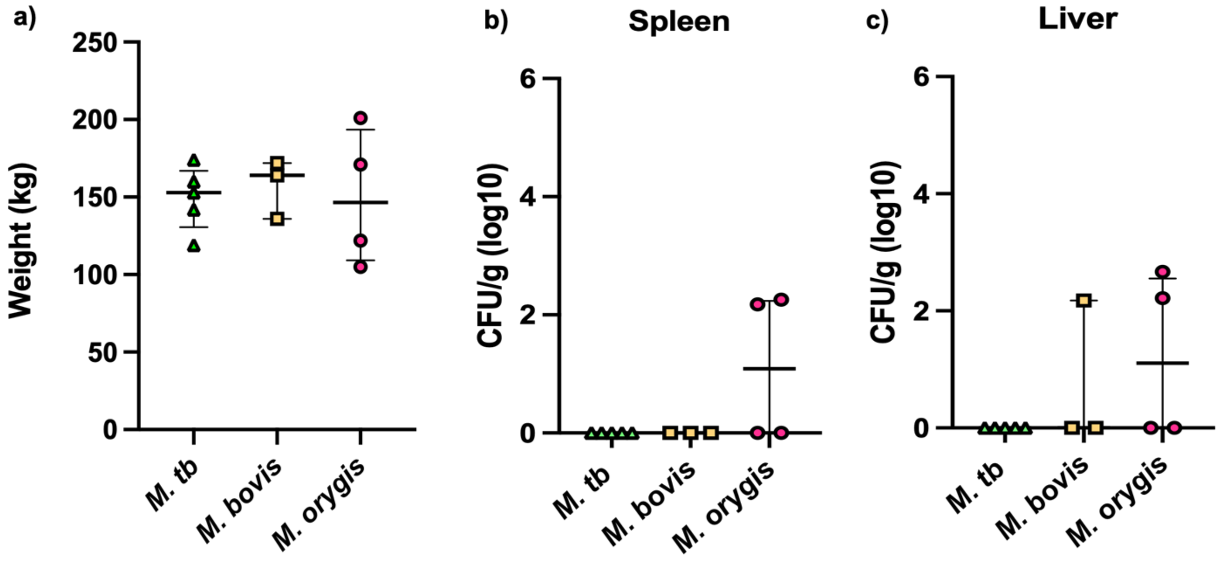

S Figure 4. Quantitative differences between MTBC infected calves.

a) Splenic bacterial burden (CFU/g) of *M. tb, M. bovis,* and *M. orygis* infected calves. 2/4 *M. orygis* calves bacteriologically confirmed (not significant). Bacteria was not observed in the spleens of *M. tb* or *M. bovis.* **b)** Hepatic bacterial burden (CFU/g) of *M. tb, M. bovis,* and *M. orygis* infected calves. 1/3 *M. bovis* calves and 2/4 *M. orygis* calves bacteriologically positive (not significant). Bacteria was not observed in any (0/5) *M. tb* infected calves. **c)** Weight of calves at necropsy (no significant differences).

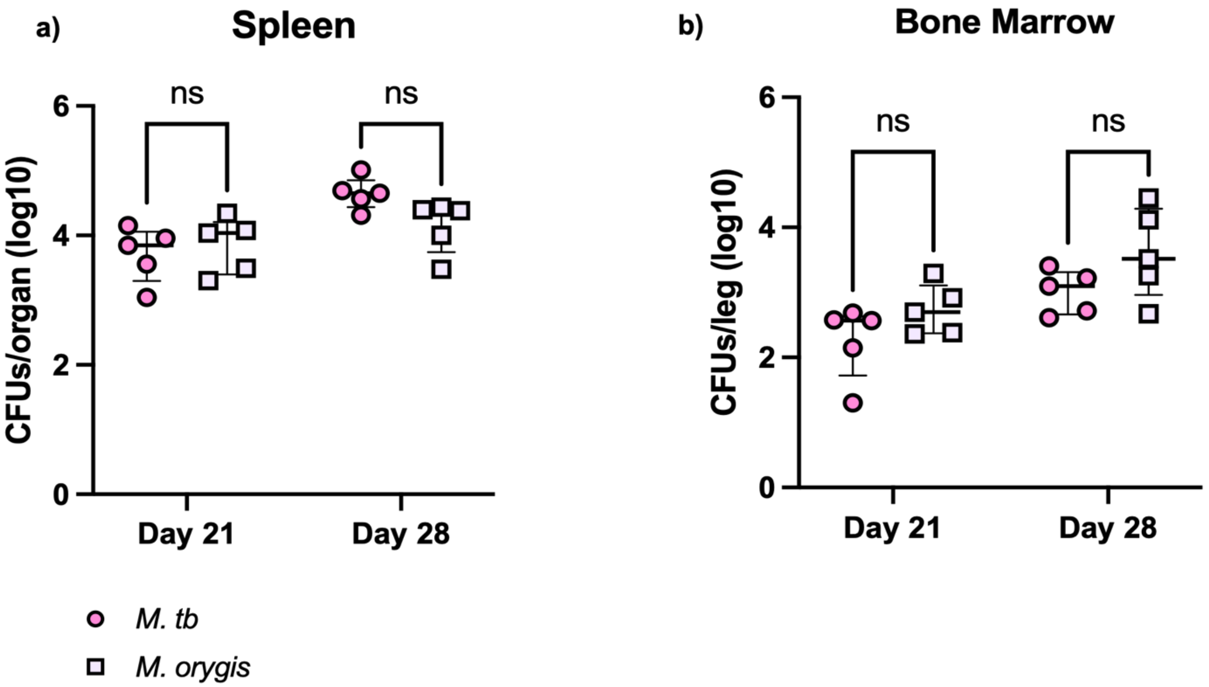

S Figure 5. Mortality following *M. orygis* infection is not caused by dissemination.
a) Splenic bacterial burden (CFU/organ) at 21- and 28-days post aerosol challenge (*M. tb* versus *M. orygis*). No significant differences observed between experimental groups regardless of timepoint assessed (2way ANOVA, $p > 0.05$). **b)** Bone marrow bacterial burden (CFUs/leg) at 21- and 28-days post aerosol challenge (*M. tb* versus *M. orygis*). No significant differences observed between experimental groups regardless of timepoint assessed (2way ANOVA, $p > 0.05$). *M. tb* and *M. orygis* experimental challenge corresponds to infection performed in Fig 2c-d.

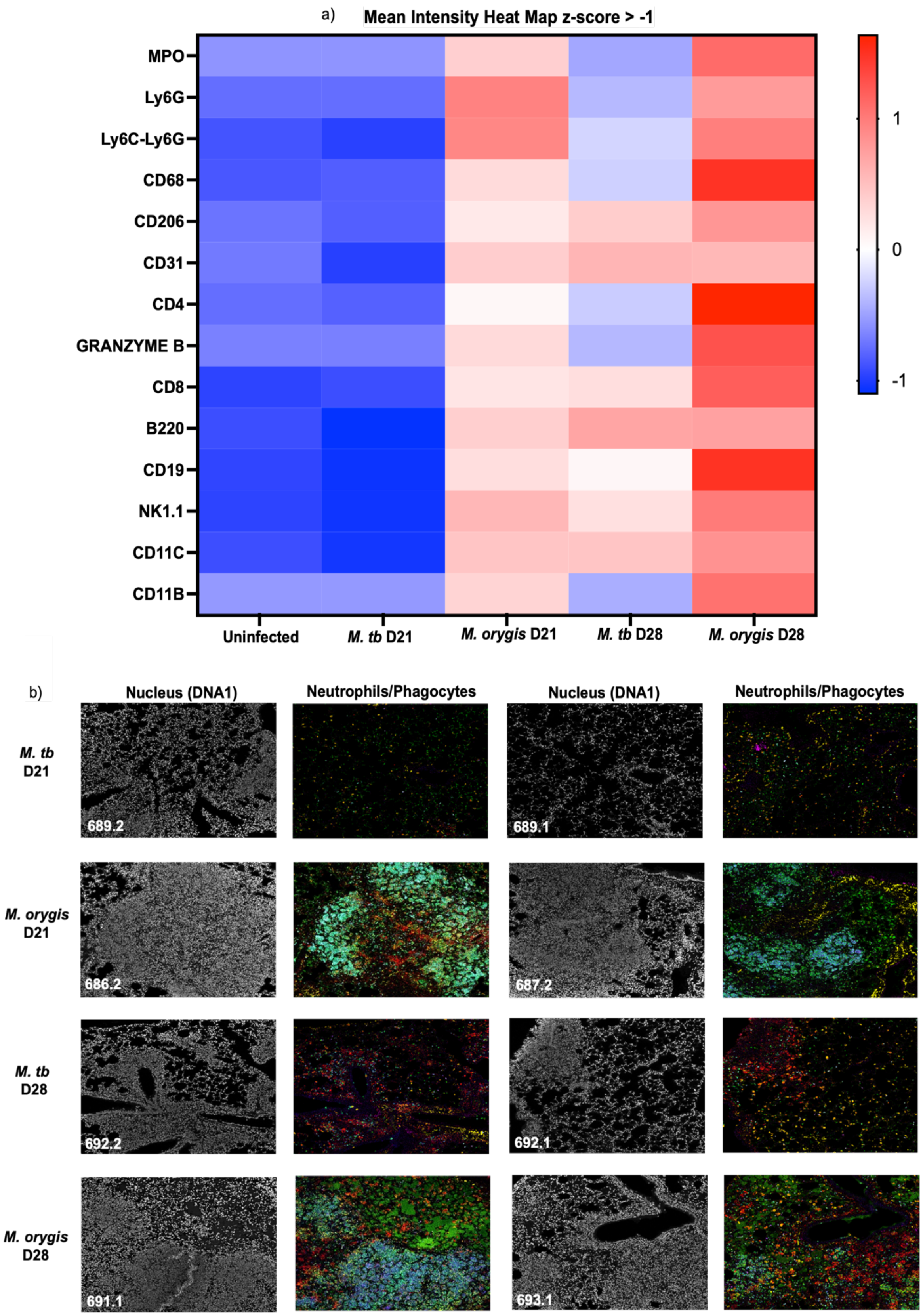
a)
Mean Intensity Heat Map z-score > -1
MPO
Ly6G
Ly6C-Ly6G
CD68
CD206
CD31
CD4
GRANZYME B
CD8
B220
CD19
NK1.1
CD11C
CD11B
Uninfected
M. tb D21
M. orygis D21
M. tb D28
M. orygis D28
1
0
-1
b)
Nucleus (DNA1)
Neutrophils/Phagocytes
Nucleus (DNA1)
Neutrophils/Phagocytes
M. tb D21
M. orygis D21
M. tb D28
M. orygis D28
689.2
689.1
686.2
687.2
692.2
692.1
691.1
693.1

c)

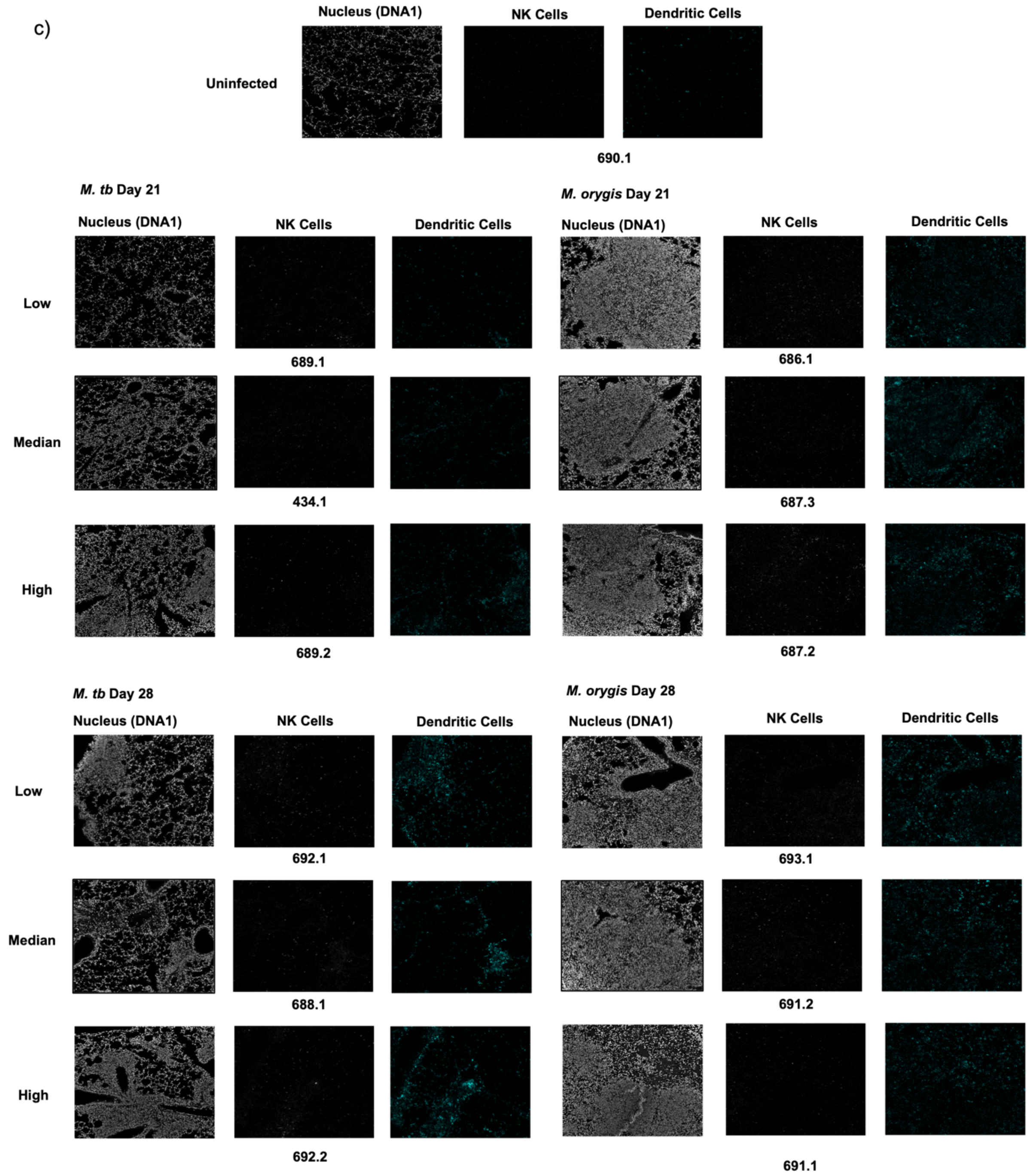
Nucleus (DNA1)
NK Cells
Dendritic Cells
Uninfected
690.1
M. tb Day 21
M. orygis Day 21
Nucleus (DNA1)
NK Cells
Dendritic Cells
Nucleus (DNA1)
NK Cells
Dendritic Cells
Low
689.1
686.1
Median
434.1
687.3
High
689.2
687.2
M. tb Day 28
M. orygis Day 28
Nucleus (DNA1)
NK Cells
Dendritic Cells
Nucleus (DNA1)
NK Cells
Dendritic Cells
Low
692.1
693.1
Median
688.1
691.2
High
692.2
691.1

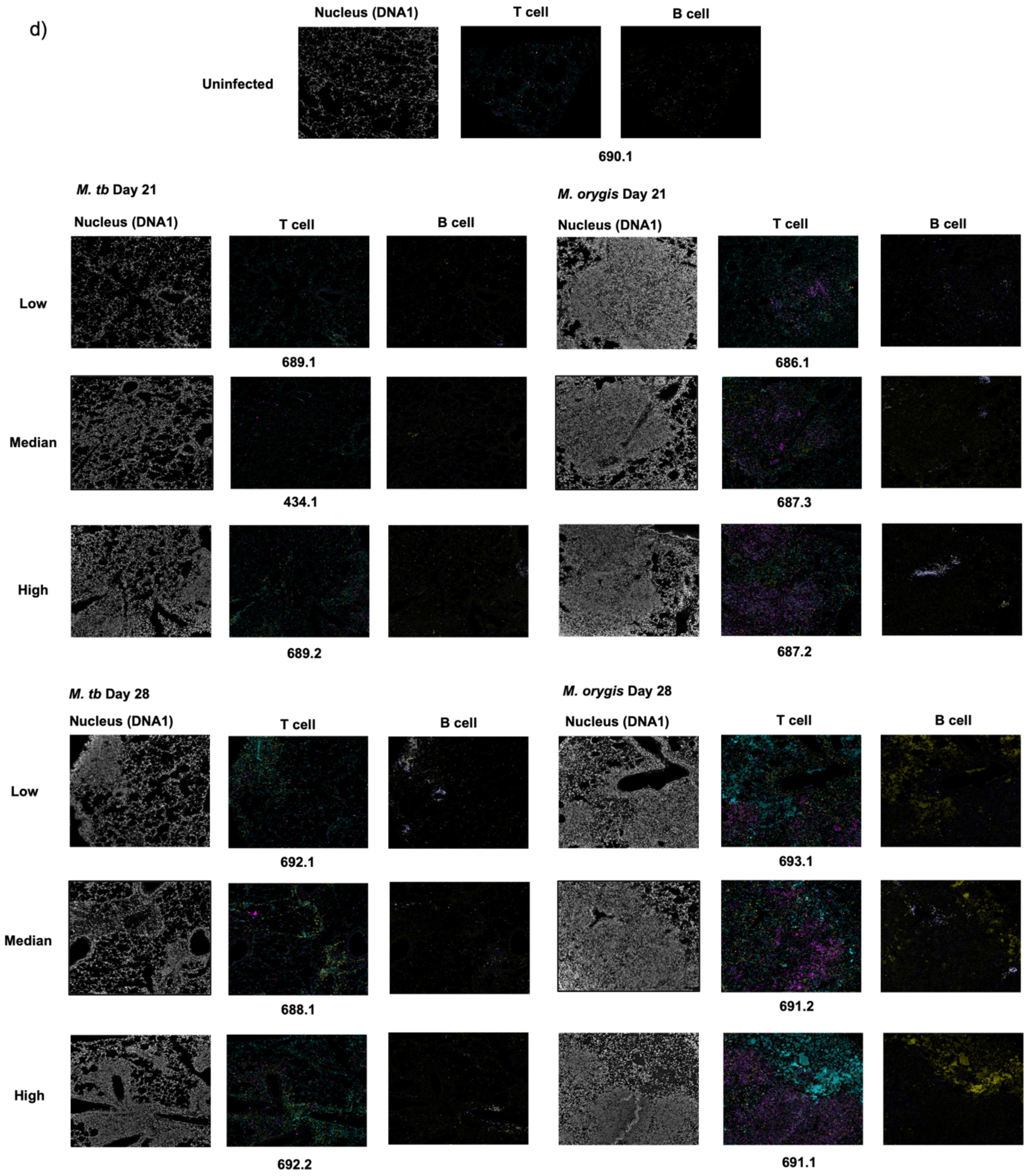

S Figure 6. SC-IMC reveals distinct differences in cell populations following MTBC infection.

a) Comparative heatmap of uninfected, *M. tb* and *M. orygis* experimental groups at pre-determined experimental endpoint (day 21 and 28) as determined by Z-score. Markers associated with neutrophil (MPO, Ly6G, Ly6C-Ly6G), macrophage (CD206), and phagocyte (CD68) populations upregulated by *M. orygis* as early as day 21. Colour intensity indicative of strength of response. Heatmap generated using GraphPad Prism v.10.0.3. **b)** SC-IMC readouts corresponding to highest (left) or lowest (right) lung

pathology score based on blinded assessment by clinical pathologist (Figure 2d). Uninfected control obtained from age-matched C57BL/6 female mouse on day 21. Red square representative of 1 mm^2 region of interest (ROI) acquired. Populations of interest: nuclear DNA (DNA1), neutrophils (MPO, Ly6G, Ly6C-Ly6G), macrophages (CD206) and phagocytes (CD68). **c)** SC-IMC readouts corresponding to the lowest, median, and highest pathology scores in each experimental group. Populations of interest: nuclear DNA (DNA1), natural killer cells (NK1.1), and dendritic cells (CD11c). **d).** SC-IMC readouts corresponding to the lowest, median, and highest pathology scores in each experimental group. Populations of interest: nuclear DNA (DNA1), helper T-cells (CD4), cytotoxic T-cells (CD8a, Granzyme B) and B-cells (CD45, CD19). Population markers and respective colours can be found in S Table 3. Images generated with MCD™ Viewer v.1.0.560.6 (post-acquisition pseudo-colouring).

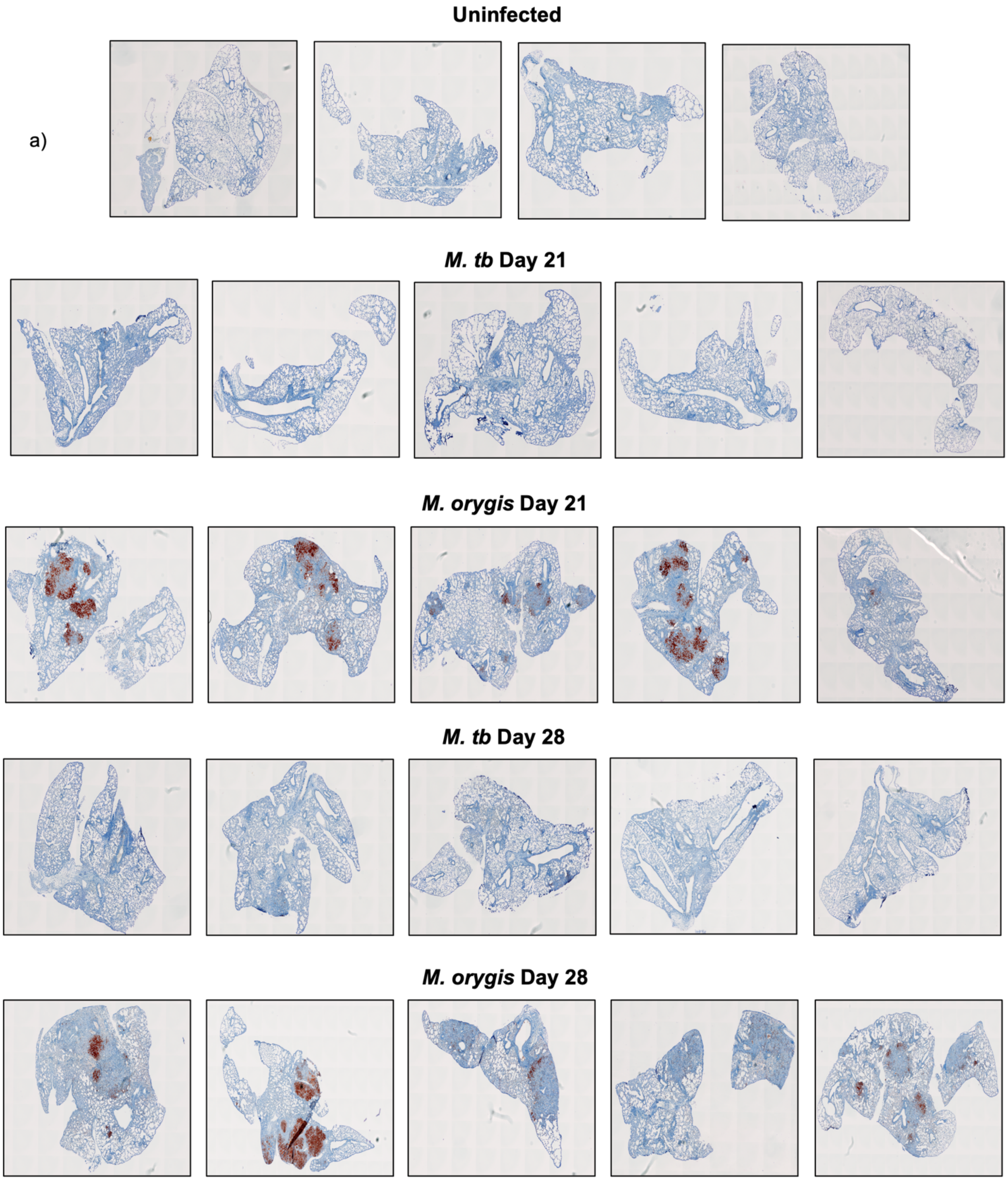
Uninfected
a)
M. tb Day 21
M. orygis Day 21
M. tb Day 28
M. orygis Day 28

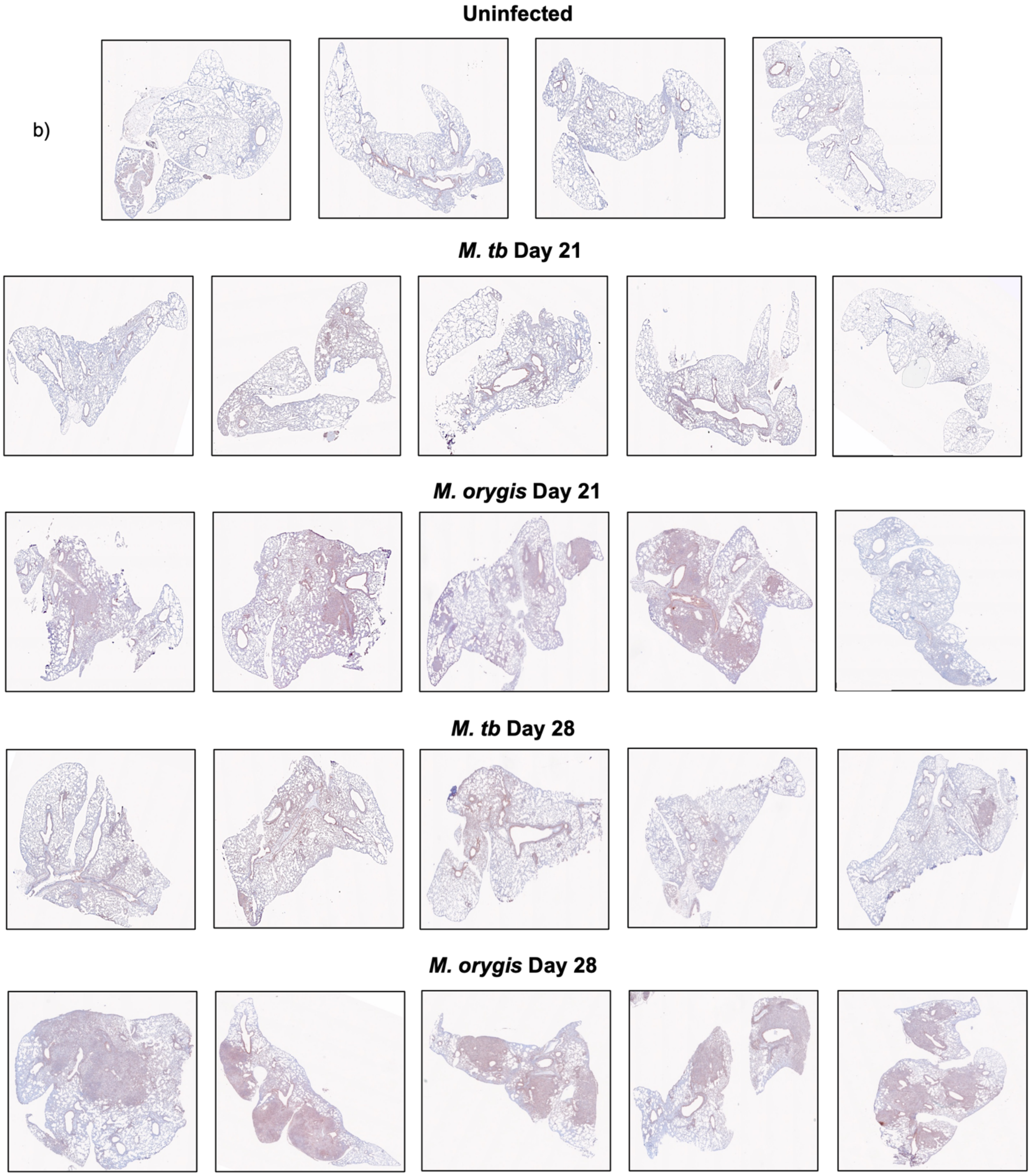
Uninfected
b)
M. tb Day 21
M. orygis Day 21
M. tb Day 28
M. orygis Day 28

c)

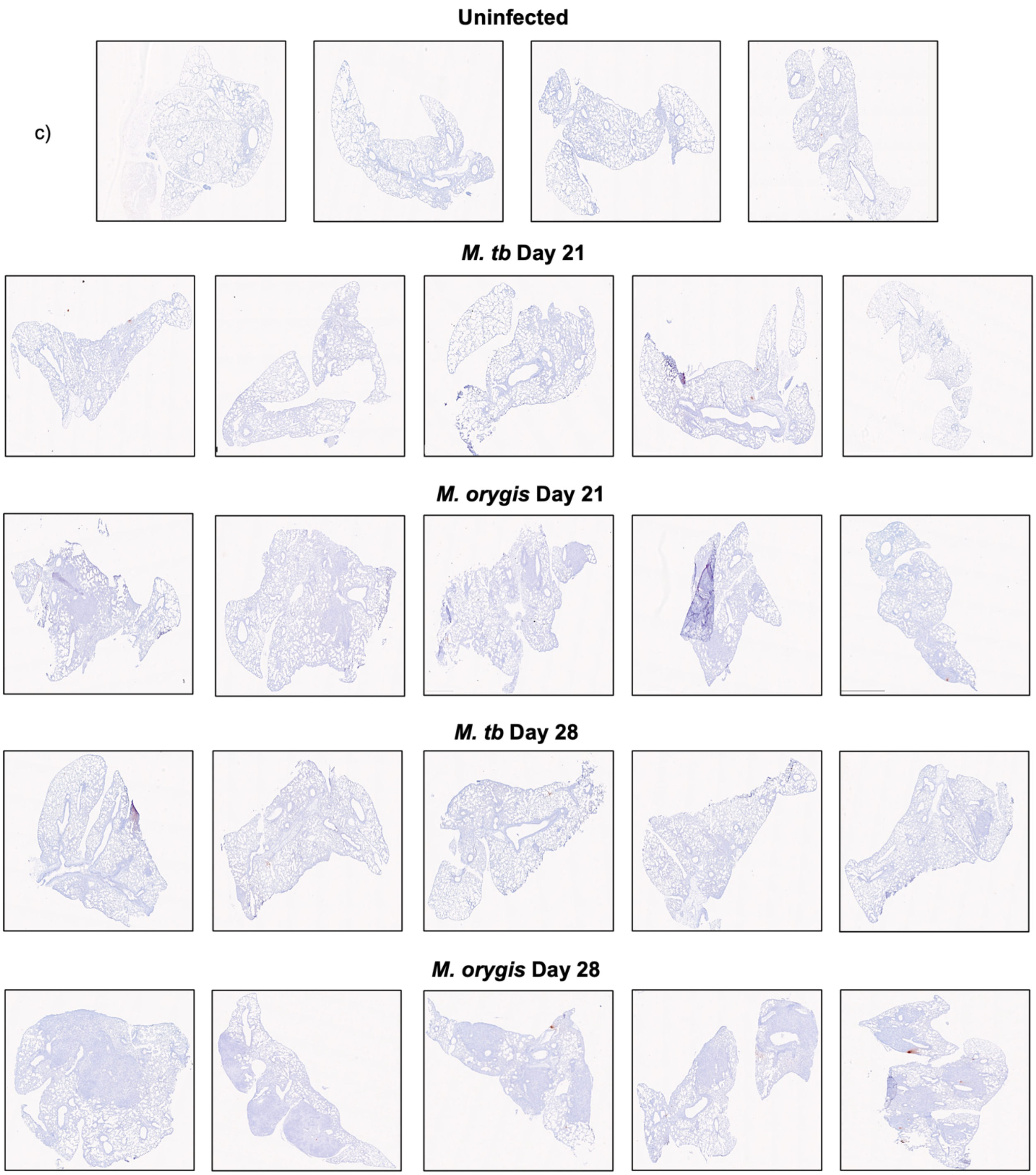

S Figure 7. IHC supports evidence of neutrophil-driven inflammatory host response to *M. orygis* challenge. a) Image of anti-Ly-6G stained right accessory lobe of lung. Neutrophil clusters highlighted in brown. Samples show marked differences between *M. orygis* infected groups at both day 21 and 28 compared to uninfected and *M. tb* infected groups. **b)** Image of anti-IL-1β stained right accessory lobe of lung. IL-1β indicated by purple-brown staining **c)** Image of anti-IL-1B stained right accessory lobe of lung.

Samples shown here correspond to the staining of sequential tissue sections of experimental groups assessed in Figure 2d and S Figure 6. Images visualized using NDP.view2 U12388-01 (Haramatsu).

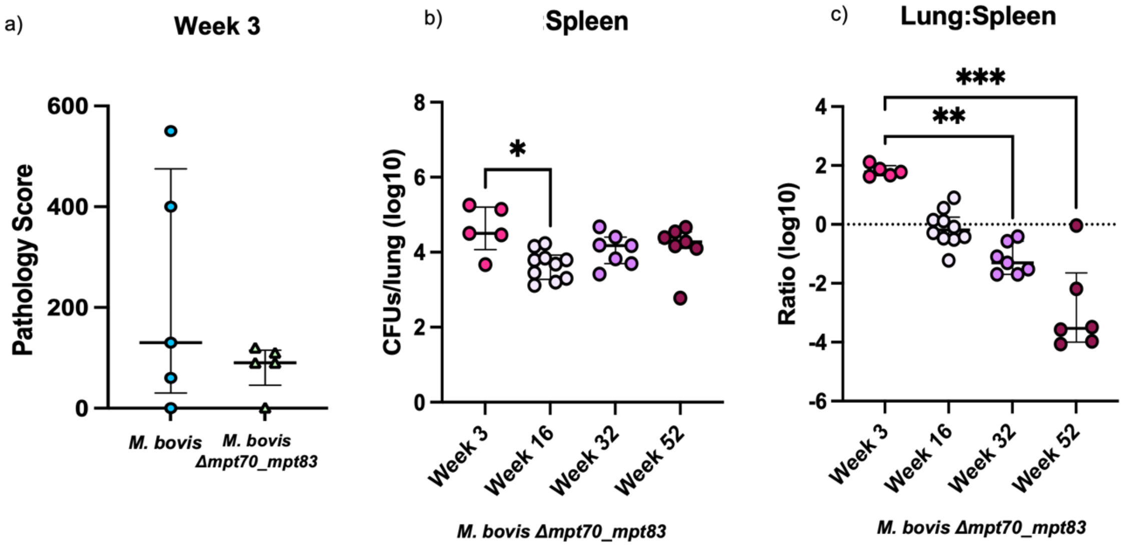

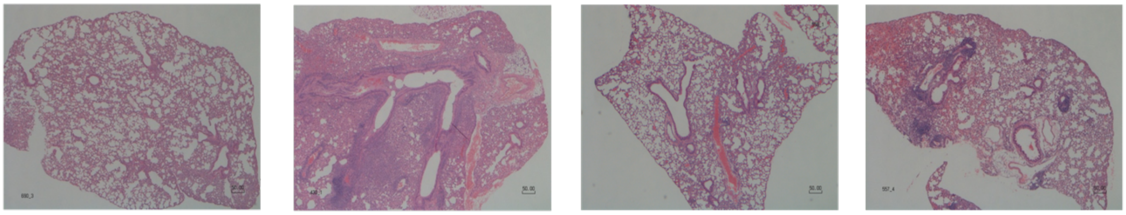

S Figure 8: *M. bovis* strains lacking MPT70 have limited lung pathology but comparable CFU in the spleen over time. a) Lung pathology score of *M. bovis* and *M. bovis ⊿mpt70_mpt83* infected mice (T= 3 weeks post-infection; not significant) **b)** Splenic bacterial burden of *M. bovis ⊿mpt70_mpt83* monitored at weeks 3, 16, 32 and 52 following aerosol infection. Spleens showed no significant reduction in bacterial burden at week 32 or 52 compared to week 3 burden (p >0.05, 2-way ANOVA). **c)** Lung:spleen ratio over time; by week 52, ratio showed complete inversion (Kruskal-Wallis test, p= 0.0001). **d)** Histopathology of right accessory lobe at week 3 and week 16 compared to uninfected control. Photos representative of overall mouse pathology.

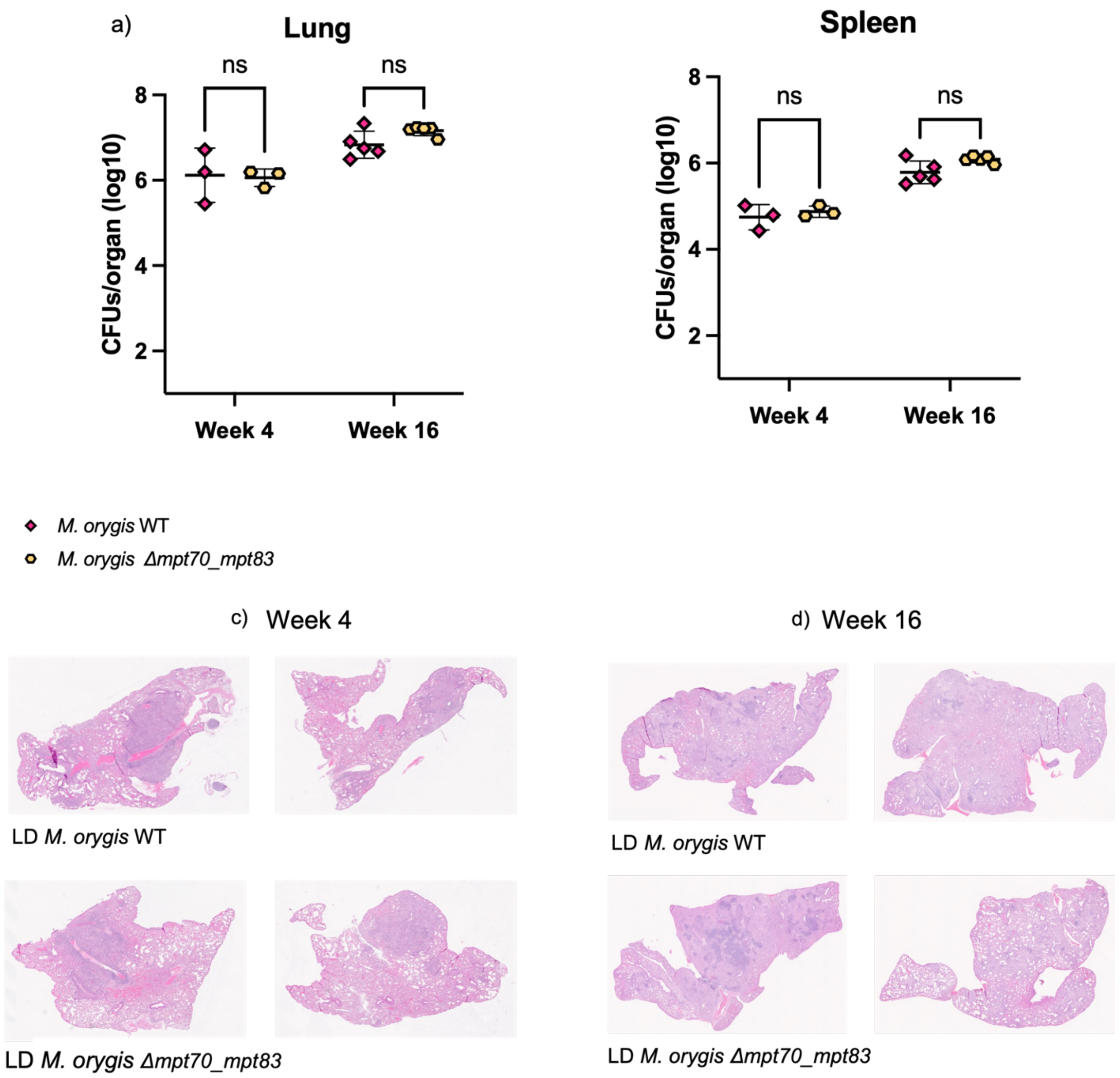

S Figure 9: Following low dose (LD) infection, *M. orygis* bacterial burden and pulmonary pathology increase over course of infection, independent of MPT70/83 status. a) Lung bacterial burden of LD *M. orygis* and LD *M. orygis Δmpt70_mpt83* at 4- and 16-weeks p.i. **b)** Splenic bacterial burden of LD *M. orygis* and LD *M. orygis Δmpt70_mpt83* at 4- and 16-weeks p.i. No significant differences between WT and isogenic groups were noted at either timepoint in either the lung nor the spleen. Both experimental groups showed an increase in bacterial burden at 16-weeks p.i. regardless of MPT70 status **b)** H&E staining of right accessory lobe of LD *M. orygis* or LD *M. orygis Δmpt70_mpt83* infected lungs, 4 weeks p.i. **c)** H&E staining of right accessory lobe of LD *M. orygis* or LD *M. orygis Δmpt70_mpt83* infected lungs, 16 weeks p.i.

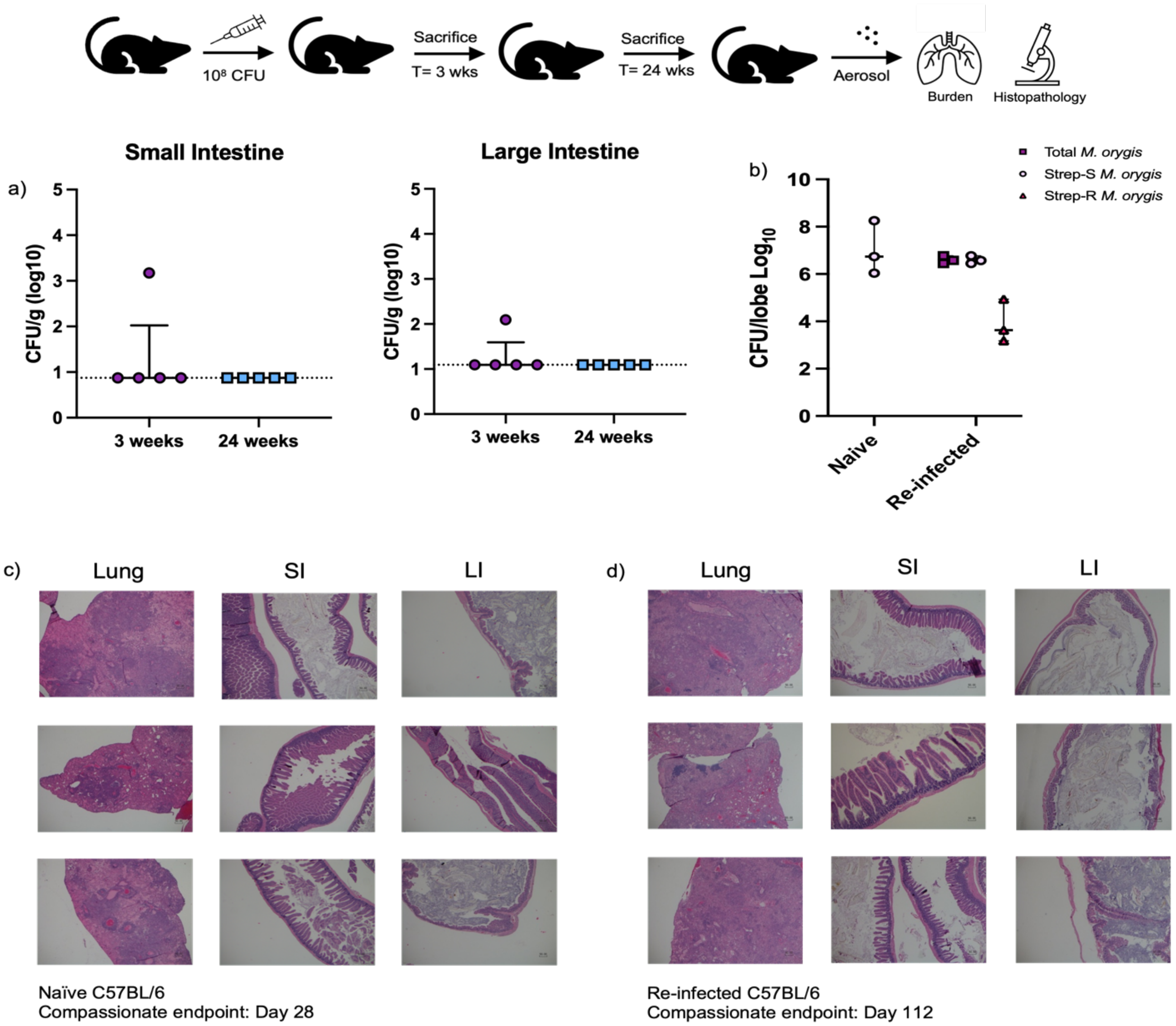

S Figure 10. Oral infection modulates but does not prevent *M. orygis* infection.

a) Bacterial burden of small and large intestines at 3- and 24-weeks post oral infection with ~10^8 CFU of streptomycin-resistant *M. orygis* (n= 5 mice per group). **b)** Bacterial burden per lobe of lung in naïve and reinfected groups at compassionate endpoint. Re-infected mice contained predominantly streptomycin susceptible *M. orygis* in the lungs (aerosol infection). A minority of bacteria recovered were streptomycin-resistant (oral infection). Total *M. orygis* recovered was comparable to bacterial burden naïve mice **(**2way-ANOVA, p> 0.99). **c)** H&E staining of lung, small intestine (SI) or large intestine (LI) at compassionate endpoint of naïve mice (day 28). **d)** H&E staining of lung, small intestine (SI) or large intestine (LI) at compassionate endpoint of reinfected mice (day 112). Both experimental groups showed extensive lung pathology, but intestinal pathology was not observed.

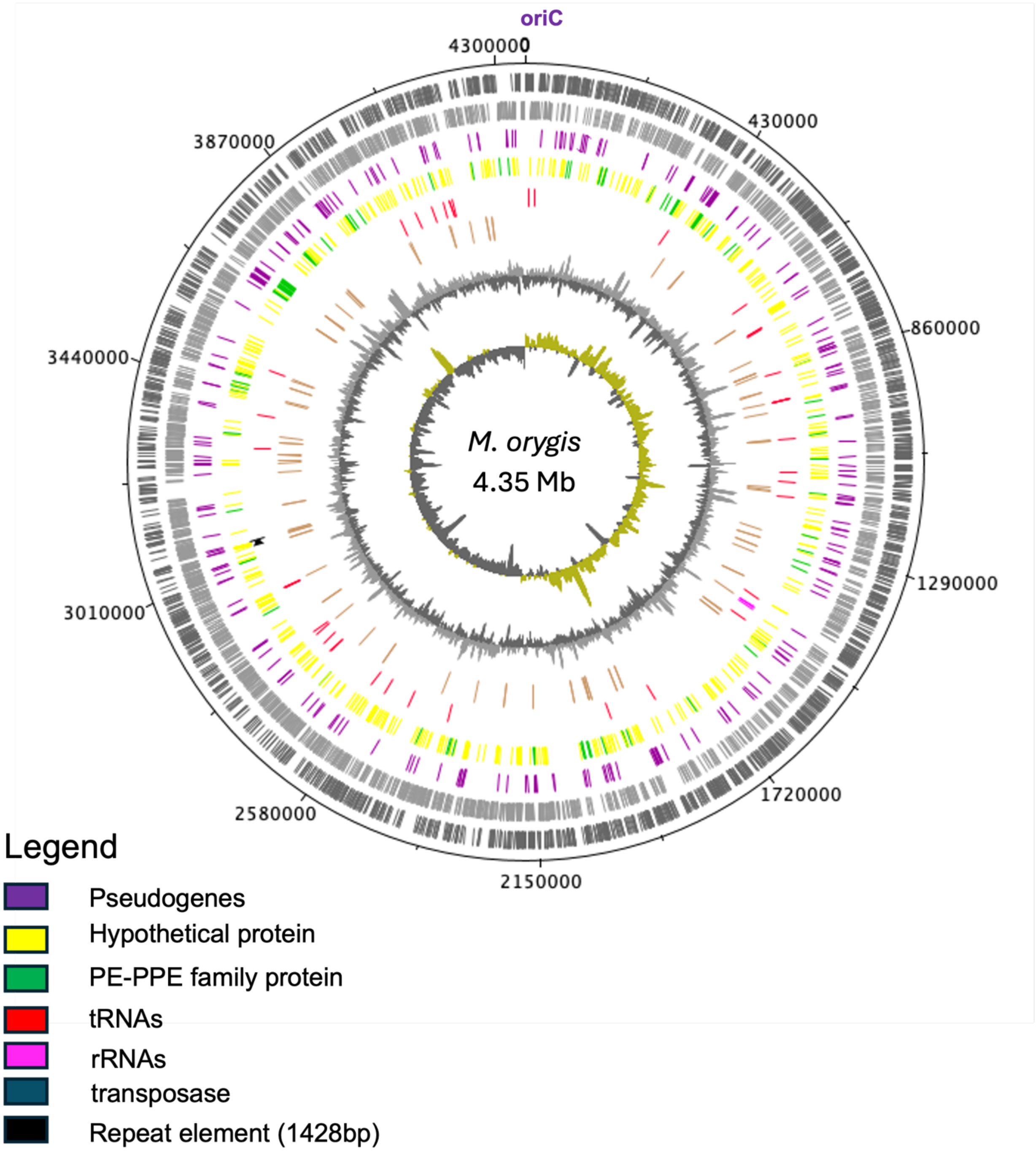

S Figure 11. Circular map of the *M. orygis* 51145 chromosome.

GC skew/histogram representing GC content. Brown= >65% GC content (values greater than the genome average); green= <65% GC content (values less than the genome average). OriC (0) represents the replication origin.

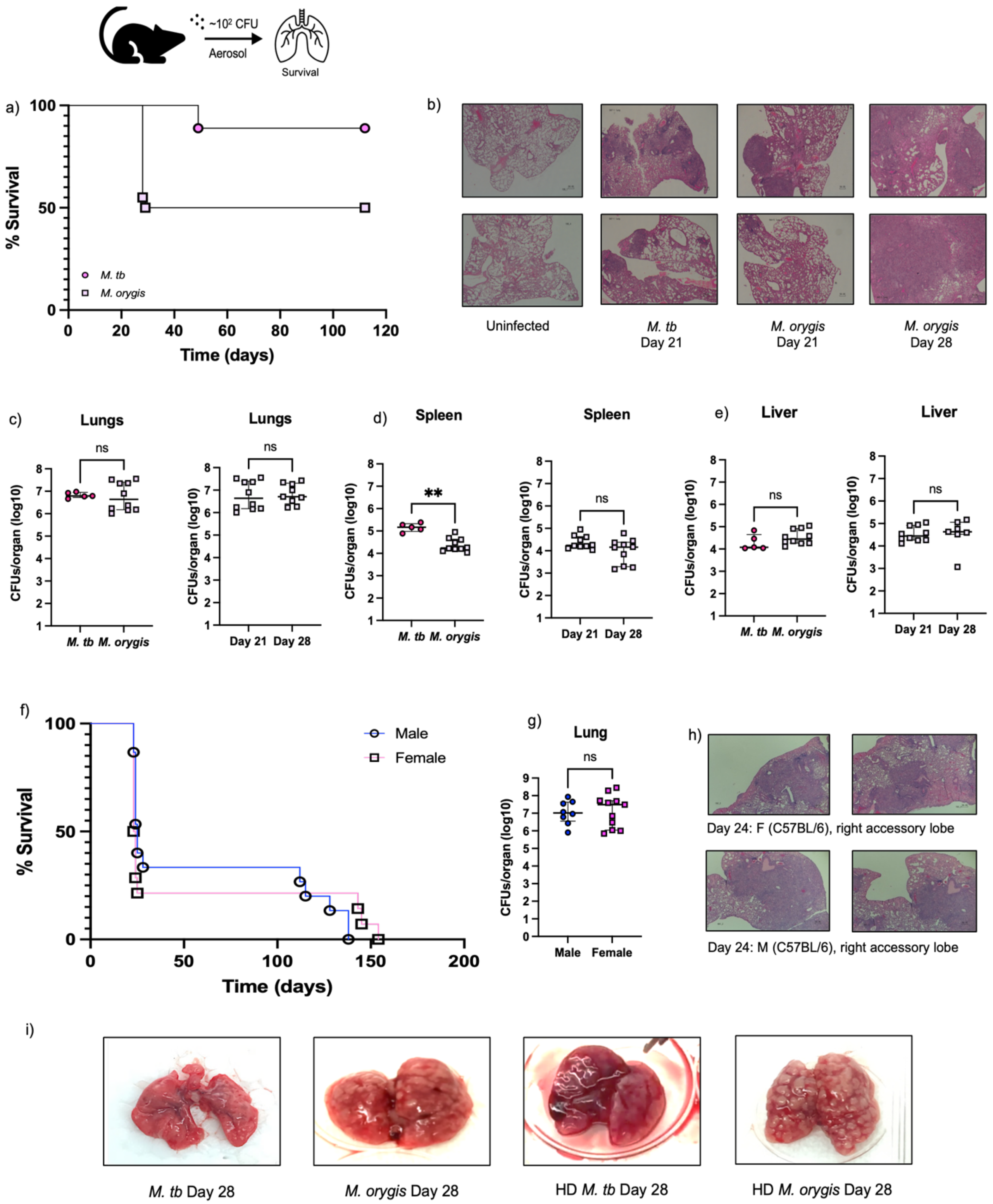
~10² CFU
Aerosol
Survival
a)
% Survival
Time (days)
M. tb
M. orygis
b)
Uninfected
M. tb Day 21
M. orygis Day 21
M. orygis Day 28
c) Lungs
Lungs
d) Spleen
Spleen
e) Liver
Liver
ns
**
CFUs/organ (log10)
M. tb M. orygis
Day 21 Day 28
f)
Male
Female
g) Lung
Male Female
h)
Day 24: F (C57BL/6), right accessory lobe
Day 24: M (C57BL/6), right accessory lobe
i)
M. tb Day 28
M. orygis Day 28
HD M. tb Day 28
HD M. orygis Day 28

S Figure 12. Characterization of *M. orygis* in the murine model.

C57BL/6 mice were exposed to ~200 CFU of either *M. tb* H37Rv or *M. orygis* 51145 (n= 2 experiments). *M. tb* H37Rv infection group n= 18 mice, *M. orygis* 51145 infection group n= 36. Three mice per experiment were tested to verify initial dose. Five mice per experiment were sacrificed at T= 21 days. **a)** Kaplan-Meier survival curve following aerosol infection. *M. orygis* median survival= 28 days. *M. tb* H37Rv median survival could not be determined by experimental endpoint (day 112). (Mantel-Cox test, p= 0.047). **b)** H&E staining of right accessory lobe of lung for *M. tb* and *M. orygis* at day 21 or compassionate endpoint (*M. orygis,* day 28). **c)** Comparison of lung bacterial burdens of *M. tb* and *M. orygis* at day 21 (left) and *M. orygis* burdens between day 21 and 28 (right). **d)** Comparison of *M. tb* and *M. orygis* spleen bacterial burdens at T= 21 (left) and *M. orygis* bacterial burdens between day 21 and 28 (right). *M. tb* showed significantly higher splenic burden compared to *M. orygis* at T= 21 days (Mann-Whitney U-test, p= 0.013). No significant difference in *M. orygis* splenic burden between day 21 and 28. **e)** Comparison of *M. tb* and *M. orygis* liver bacterial burden at T= 21 days (left) and *M. orygis* bacterial burdens between day 21 and 28 (right). **f)** Kaplan-Meier survival curve following aerosol infection of male or female C57BL/6 (n= 18 mice per group). Median survival of males= 25 days; median survival of females= 23.5 days (Mantel-Cox test, p= 0.993). **g)** Lung bacterial burdens at compassionate endpoint. Regardless of when mice succumbed to infection, bacterial burden showed no significant difference (Mann-Whitney test, p= 0.778). **h)** Comparative lung pathology at compassionate endpoint. Right accessory lobe stained with H&E. Lungs presented harvested from male or female mouse that succumbed to infection on same day (T= 24 days). **i)** Macroscopic pathology of *M. tb* and *M. orygis* infected mice at day 28 at standard (left) or high dose (right). Standard dose defined as ~150-300 CFU; high dose defined as ~1300 CFU.

Supplemental Tables

S Table 1. Lesion presentation. Infected calves positive for gross lesions in the lungs, lymph nodes, spleen or liver at necropsy. TBLN: tracheobronchial lymph nodes; MLN= mediastinal lymph nodes. Lungs considered positive if lesions were detected in any lobe.

	M. tb	***M. bovis***	***M. orygis***
**Lungs**	3/5 (60%)	3/3 (100%)	4/4 (100%)
**TBLN**	0/5 (0%)	3/3 (100%)	4/4 (100%)
**MLN**	0/5 (0%)	3/3 (100%)	4/4 (100%)
**Spleen**	0/5 (0%)	0/3 (0%)	0/4 (0%)
**Liver**	0/5 (0%)	0/3 (0%)	2/4 (50%)

S Table 2. Semiquantitative scoring scale of histological parameters. For inflammation intensity, vascular damages, and pulmonary edema fractions of points were also allowed. Very mild= 0.5, mild to moderate= 1.5, and moderate to marked= 2.5

**Parameter**	**Score**			
**Quality control**	**0**	**1**	**2**	**3**
**Tissue quality**	Inadequate	Good	Very good	Excellent
**Distribution of lesions**	Bronchial/Peribronchial	Parenchymal		
**% affected area**	0-100%			
**Inflammation intensity**	**0**	**1**	**2**	**3**
**Bronchial/endobronchial**	Normal/absent	Mild	Moderate	Marked
**Peribronchial**	Normal/absent	Mild	Moderate	Marked
**Perivascular**	Normal/absent	Mild	Moderate	Marked
**Interstitial**	Normal/absent	Mild	Moderate	Marked
**Pleural**	Normal/absent	Mild	Moderate	Marked
**Intra-alveolar**	Normal/absent	Mild	Moderate	Marked
**Inflammatory cellular infiltrate**	N/A	A	C	AC
**Type of cellular infiltrate**	Not applicable	Acute (neutrophils)	Chronic (lymphohistiocytic)	A and C
**Vascular damages**	0	1	2	3
**Hemorrhages**				
**Endothelialitis**				
**Capillary vascular congestion**	Absent	Mild	Moderate	Marked
**Pulmonary edema**	**0**	1	2	3
**Pulmonary edema**	Absent	Mild	Moderate	Marked

S Table 3. Conjugated antibodies used in immune cell profiling and their respective metal-tags. Colours correspond to Fig. 2e pseudo-colouring. * indicates markers shown in Fig. 2e and S Fig 5c. DNA1 included as nuclear control.

**Cell Population**	**Metal-Tag**	**Conjugated Antibody**	**Colour**
Nuclei	191Ir	DNA1*	White*
Neutrophils	151Eu	Ly6G*	Blue*
Neutrophils	176Yb	Ly6C-Ly6G*	Pink*
Neutrophils (major)<br>Monocytes (minor)	163Dy	Myeloperoxidase (MPO)*	Green*
Phagocytes	165Ho	CD68*	Red*
Macrophages	169Tm	CD206*	Yellow*
Helper T-cells	164Dy	CD4	Teal
Cytotoxic T-cells	155Gd	Granzyme B	Pink
Cytotoxic T-cells	153Eu	CD8a	Yellow
B-cells	142Nd	CD45 (B220)	Blue
B-cells	161Dy	CD19	Yellow
Natural killer cells (NK)	145Nd	NK1.1	White
Dendritic cells (DC)	154Sm	CD11c	Teal

S Table 4. Characteristics of *Mycobacterium tuberculosis* complex genomes

Annotation	Genbank GCA_000195955.2	RefSeq GCF_000195955.2	H37Rv-1, Chitale et al. 2022	Genbank GCA_018305025.1	RefSeq GCF_018305025.1	this study / GenBank GCA_015265495.2
**Assembly level**	Chromosome		Chromosome	Scaffold		Chromosome
**Genome Size (bp)**	4411532		4417942	4377551		4352140
**GC content (%)**	65.5		65.6	65.5		65.6
**Genes**	4111	4008	4157	4109	4187	4085
**CDSs (coding)**	4018	3906	3955	3866	3864	3778
**Pseudogenes (total)**	13	30	151	192	272	256
**Transposases (total)**	56	56	74	72	74	75
**tRNA**	45	45	45	45	45	45
			**PE-family**			
**Annotated as PE family (pseudogenes)**	100 (2)	94 (2)	100 (15)	105 (20)	109 (28)	97 (30)
**Hmmsearch (PF00934)**	91	90	84	77	77	66
**Hmmsearch (PF000934), prokka annotation**	89		89	70		89
			**PPE-family**			
**Annotated as PPE family (pseudogenes)**	69 (2)	65 (2)	68 (6)	76 (9)	75 (11)	64 (13)
**Hmmsearch (PF00823)**	66	63	64	63	63	51
**Hmmsearch (PF00823), prokka annotation**	66		67	49		65

S Table 5. Bacterial strains used in virulence studies. * indicates use in gavage infection; Strep-R = streptomycin-resistant. All *M. bovis* isogenic strains are derived from *M. bovis* Ravenel; all *M. orygis* isogenic strains are derived from *M. orygis* 51145.

**Wildtype strains**	**Engineered strains**
M. orygis 51145 (*M. or;* reference)	*M. orygis rpsL* K43R (Strep-R)*
M. orygis 2019-352 (clinical isolate)	*M orygis ΔesxA*
M. bovis Ravenel (*M. bo*)	*M. orygis Δmpt70_mpt83*
M. bovis AF2122/97 (*M. bo,* reference)	*M. bovis ΔesxA*
M. tuberculosis H37Rv (*M. tb;* reference)	*M. bovis Δmpt70_mpt83*
M. tuberculosis Erdman	*M. bovis Δmpt70*
M. bovis BCG Russia	
M. bovis BCG Danish	

S Table 6. Lung pathology scoring criteria. Adapted from Dr. Jeffery Chen, University of Saskatchewan; Waters et al. 2014

Score	Description
**0**	No gross lesions
**1**	< 10 lesions AND/OR<br>All lesions < 10 mm in diameter
**2**	6-10 lesions AND/OR<br>Rare (< 2) lesions between 10-20 mm in diameter
**3**	11-20 lesions AND/OR<br>Occasional (3-5) lesions between 10-20 mm in diameter
**4**	> 20 lesions AND/OR<br>Frequent (> 5) between 10-20 mm in diameter
**5**	Numerous and coalescing lesions AND/OR<br>> 50% lesions > 10 mm<br>> Identification of any lesions > 20 mm

S Table 7. Clinical assessment of mice. If behaviour/appearance reached score of 3 or respiration/dehydration reached score of 2, animal was euthanized. If body condition score (BCS) is < 2 or body weight loss > 20%, animal was euthanized.

Behavior (B)

Clinical Sign	Score
BAR (Bright, alert and responsive)	0
Active but hunched posture	1
Less active when observed outside of cage but active when stimulated	2
Inactive even when stimulated (no respond to touch, no righting reflex)	3

Appearance (A)

Clinical Sign	Score
Normal (hair coat and posture, eyes open)	0
Slightly hunched, piloerection, eyes partially closed	1
Moderately hunched, piloerection, eyes moderately closed, pale mucous membranes	2
Ruffled coat, very hunched, eyes completely closed, pale mucous membranes	3

Respiration (R)

Clinical Sign	Score
Normal	0
Increased respiratory rate, mild abdominal breathing	1
Dyspnea (severely increased respiratory rate, open-mouth and abdominal breathing)	2

Dehydration (D)

Clinical Sign	Score
Normal (pinching the skin over the shoulder blades, the skin will quickly return to its original shape)	0
Moderate (Delay in skin return to its normal position after pinching the skin over the shoulder blades)	1
Severe (sunken eyes, skin stay bunched up)	2

Body Condition (BC)	Score
Mouse is emaciated; skeletal structure extremely prominent, vertebrae distinctly segmented	1
Mouse is under-conditioned; segmentation of vertebral column evident, dorsal pelvic bones are easily palpable	2
Normal; mouse is well-conditioned. Vertebrae and dorsal pelvis not prominent but palpable with light pressure	3
Mouse is over-conditioned; vertebrae palpable only with firm pressure	4
Mouse is obese; bone structure disappears under flesh and subcutaneous fat	5

S Table 8. Genomic corrections following Illumina HiSeq

Position	PacBio	Illumina	Codon change	Sanger	Locus_Tag	Gene name
**2017392**	T	C	p.Met507Thr	**C**	RJtmp_001858	eccC5
**577991**	TT	T	.	**T**	RJtmp_000510	mshA
**2093004**	T	G	p.Asp38Ala	**G**	RJtmp_001924	Rv1856c
**3161714**	CC	C	.	**C Del**	gene_RJtmp_002996	Rv2905 (lppw)
**3161737**	GTC	T	.	G Del	gene_RJtmp_002996	Rv2905 (lppw)
**3161754**	A	G	.	A Del	gene_RJtmp_002996	Rv2905 (lppw)
**3161764**	TT	T	.	T Del	gene_RJtmp_002996	Rv2905 (lppw)
**3161772**	AA	A	.	A Del	gene_RJtmp_002996	Rv2905 (lppw)
**3161779**	TT	T	.	T Del	gene_RJtmp_002996	Rv2905 (lppw)
**3161790**	CC	C	.	C Del	gene_RJtmp_002996	Rv2905 (lppw)
**3161799**	TTT	T	.	T Del	gene_RJtmp_002996	Rv2905 (lppw)
**3161811**	AA	A	.	A Del	gene_RJtmp_002996	Rv2905 (lppw)
**3161818**	CC	C	.	C Del	gene_RJtmp_002996	Rv2905 (lppw)
**3161828**	AA	A	.	A Del	gene_RJtmp_002996	Rv2905 (lppw)
**3161834**	GG	G	.	G Del	gene_RJtmp_002996	Rv2905 (lppw)
**3161843**	TT	T	.	T Del	gene_RJtmp_002996	Rv2905 (lppw)
**3161850**	AA	A	.	A Del	gene_RJtmp_002996	Rv2905 (lppw)
**3161881**	CC	C	.	C Del	gene_RJtmp_002996	Rv2905 (lppw)
**3161889**	AA	A	.	A Del	gene_RJtmp_002996	Rv2905 (lppw)
**3161898**	CGGA	GG	.	C Del	gene_RJtmp_002996	Rv2905 (lppw)
**3161929**	CC	C	.	C Del	gene_RJtmp_002996	Rv2905 (lppw)
**3161940**	CC	C	.	C Del	gene_RJtmp_002996	Rv2905 (lppw)
**3161947**	CC	C	.	C Del	gene_RJtmp_002996	Rv2905 (lppw)
**3161967**	AA	A	.	A Del	gene_RJtmp_002996	Rv2905 (lppw)
**3161979**	AA	A	.	A Del	gene_RJtmp_002996	Rv2905 (lppw)
**3162155**	TT	T	.	**T Del**	gene_RJtmp_002997	Rv2906c(trmD)
**3162210**	GTC	T	.	**T Del**	gene_RJtmp_002997	Rv2906c(trmD)
**3162228**	AA	A	.	**A Del**	gene_RJtmp_002997	Rv2906c(trmD)
**3162245**	GG	G	.	**G Del**	gene_RJtmp_002997	Rv2906c(trmD)
**3162282**	CC	C	.	**C Del**	gene_RJtmp_002997	Rv2906c(trmD)
**3162291**	GG	G	.	**G Del**	gene_RJtmp_002997	Rv2906c(trmD)
**3162299**	CC	C	.	**C Del**	gene_RJtmp_002997	Rv2906c(trmD)
**3162303**	GGC	G	.	**G Del**	gene_RJtmp_002997	Rv2906c(trmD)
**3162311**	TT	T	.	**T Del**	gene_RJtmp_002997	Rv2906c(trmD)

S Data 1. (separate file)
MTBC secretomic data (spectral counts)

S Data 2. (separate file)
MTBC secretomic data (Z-scores)

S Data 3. (separate file)
SC-IMC data

SI References

1. S. C. Duffy, *et al.*, Establishment of persistent enteric mycobacterial infection following streptomycin pre-treatment. *Gut Pathogens* **15**, 46 (2023).

2. K. C. Murphy, *et al.*, ORBIT: a New Paradigm for Genetic Engineering of Mycobacterial Chromosomes. *mBio* **9**, e01467-18 (2018).

3. E. Karimi, *et al.*, Single-cell spatial immune landscapes of primary and metastatic brain tumours. *Nature* **614**, 555–563 (2023).

4. W. R. Waters, *et al.*, Virulence of Two Strains of *Mycobacterium bovis* in Cattle Following Aerosol Infection. *Journal of Comparative Pathology* **151**, 410–419 (2014).

5. P. Chitale, *et al.*, A comprehensive update to the Mycobacterium tuberculosis H37Rv reference genome. *Nat Commun* **13**, 7068 (2022).

6. S. R. Eddy, Accelerated Profile HMM Searches. *PLOS Computational Biology* **7**, e1002195 (2011).

7. T. Seemann, Prokka: rapid prokaryotic genome annotation. *Bioinformatics* **30**, 2068–2069 (2014).

8. L. B. Harrison, V. Kapur, M. A. Behr, An imputed ancestral reference genome for the Mycobacterium tuberculosis complex better captures structural genomic diversity for reference-based alignment workflows. *Microbial Genomics* **10**, 001165 (2024).

9. H. S. Clemmensen, *et al.*, Rescuing ESAT-6 Specific CD4 T Cells From Terminal Differentiation Is Critical for Long-Term Control of Murine Mtb Infection. *Front Immunol* **11**, 585359 (2020).

10. H. S. Clemmensen, *et al.*, *In Vivo* Antigen Expression Regulates CD4 T Cell Differentiation and Vaccine Efficacy against Mycobacterium tuberculosis Infection. *mBio* **12**, e00226-21 (2021).

11. K. Dijkman, *et al.*, A protective, single-visit TB vaccination regimen by co-administration of a subunit vaccine with BCG. *NPJ Vaccines* **8**, 66 (2023).

12. S. B. Rufai, *et al.*, Complete Genome Sequence of Mycobacterium orygis Strain 51145. *Microbiology Resource Announcements* **10**, 10.1128/mra.01279-20 (2021).

13. R. Brosch, *et al.*, A new evolutionary scenario for the Mycobacterium tuberculosis complex. *Proc Natl Acad Sci U S A* **99**, 3684–3689 (2002).

14. S. Mostowy, *et al.*, Revisiting the Evolution of Mycobacterium bovis. *J Bacteriol* **187**, 6386–6395 (2005).

15. B. Saïd-Salim, S. Mostowy, A. S. Kristof, M. A. Behr, Mutations in Mycobacterium tuberculosis Rv0444c, the gene encoding anti-SigK, explain high level expression of MPB70 and MPB83 in Mycobacterium bovis. *Molecular Microbiology* **62**, 1251–1263 (2006).

16. S. C. Duffy, *et al.*, Reconsidering Mycobacterium bovis as a proxy for zoonotic tuberculosis: a molecular epidemiological surveillance study. *Lancet Microbe* **1**, e66–e73 (2020).

PNAS

Supporting Information for

Virulence hierarchies within the Mycobacterium tuberculosis complex

Short title: Differential virulence of the MTBC

Sarah N. Danchuk[1,2,3], Shannon C. Duffy[4], Jaryd Sullivan[5], Syed Beenish Rufai[6,7], Fiona A. McIntosh[2,3], Andréanne Lupien[1,2,3,8], Luke B. Harrison[2,8], Hojjat Ghasemi Goojani[2,3], Lorne Taylor[2], Yuhong Wei[9], Philippe Joubert[10], Rasmus Mortensen[11], Jeffrey M. Chen[12], Nirajan Niroula[12], Robin Stevens[12], Carla Norleen[12], Vivek Kapur[13], Marcel A. Behr*[1,2,3,8]

1. Department of Microbiology and Immunology, McGill University, Montreal, QC, Canada
2. Research Institute of the McGill University Health Centre, Montreal, QC, Canada
3. McGill International Tuberculosis Centre, Montreal, QC, Canada
4. Department of Epidemiology of Microbial Diseases, Yale School of Public Health, New Haven, CT, USA
5. Department of Molecular Biology and Centre for Computational and Integrative Biology, Massachusetts General Hospital, Boston, MA, USA
6. Department of Neuroscience, University of Lethbridge, Alberta, Canada
7. Department of Biochemistry and Medical Genetics, University of Manitoba, Winnipeg, Canada
8. Department of Medicine, McGill University Montreal, Montreal, QC, Canada
9. Goodman Cancer Institute, McGill University, Montreal, QC, Canada
10. Institut Universitaire de Cardiologie et de Pneumologie de Québec-Laval University, Quebec City, QC, Canada
11. Center for Vaccine Research, Department of Infectious Disease Immunology, Statens Serum Institut, Copenhagen, Denmark
12. Vaccine and Infectious Disease Organization (VIDO), University of Saskatchewan, Saskatoon, SK, Canada
13. Department of Animal Science and the Huck Institutes of the Life Sciences, The Pennsylvania State University, University Park, PA, USA

Correspondence to: Prof Marcel A. Behr, Research Institute of the McGill University Health Centre, Montreal, QC, H4A 3J1, Canada, marcel.behr@mcgill.ca

This PDF file includes:

Supplemental Methods
Supplemental Results
S Figures 1 to 12
S Tables 1 to 8
Legends for S Data 1 to 3
SI References

Other supporting materials for this manuscript include the following:

S Data 1 to 3
